# SARS-CoV-2 tropism to intestinal but not gastric epithelial cells is defined by limited ACE2 expression

**DOI:** 10.1016/j.stemcr.2024.03.008

**Published:** 2024-04-25

**Authors:** Mindaugas Paužuolis, Diana Fatykhova, Boris Zühlke, Torsten Schwecke, Mastura Neyazi, Pilar Samperio-Ventayol, Carmen Aguilar, Nicolas Schlegel, Simon Dökel, Markus Ralser, Andreas Hocke, Christine Krempl, Sina Bartfeld

**Affiliations:** 1 Research Centre for Infectious Diseases, Institute for Molecular Infection Biology, Julius Maximilians Universität Würzburg, Würzburg, Germany; 2 Department of Infectious Diseases, Respiratory Medicine and Critical Care, Charité - Universitätsmedizin Berlin, Corporate Member of Freie Universität Berlin and Humboldt-Universität zu Berlin, Berlin, Germany; 3 Institute of Biochemistry, Charité - Universitätsmedizin Berlin, Berlin, Germany; 4 Core Facility for High-Throughput Mass Spectrometry, Institute of Biochemistry, Charité - Universitätsmedizin Berlin, Berlin, Germany; 5 Si-M/‘Der Simulierte Mensch’, Technische Universität Berlin and Charité–Universitätsmedizin Berlin, Berlin, Germany; 6 Department of Medical Biotechnology, Institute of Biotechnology, Technische Universität Berlin, Berlin, Germany; 7 Department of General, Visceral, Transplant, Vascular and Pediatric Surgery, University Hospital Würzburg, Würzburg, Germany; 8 Institute of Veterinary Pathology, Freie Universität Berlin, Berlin, Germany; 9 The Francis Crick Institute, Molecular Biology of Metabolism Laboratory, London, UK; 10 The Wellcome Centre for Human Genetics, Nuffield Department of Medicine, University of Oxford, Oxford, UK; 11 Institute for Virology and Immunobiology, Julius Maximilian University of Würzburg, Würzburg, Germany

**Keywords:** organoids, gastrointestinal epithelium, SARS-CoV-2, infection model, ACE2, intestine, stomach

## Abstract

Severe acute respiratory syndrome coronavirus 2 (SARS-CoV-2) infection primarily affects the lung but can also cause gastrointestinal (GI) symptoms. *In vitro* experiments confirmed that SARS-CoV-2 robustly infects intestinal epithelium. However, data on infection of adult gastric epithelium are sparse and a side-by-side comparison of the infection in the major segments of the GI tract is lacking. We provide this direct comparison in organoid-derived monolayers and demonstrate that SARS-CoV-2 robustly infects intestinal epithelium, while gastric epithelium is resistant to infection. RNA sequencing and proteome analysis pointed to angiotensin-converting enzyme 2 (ACE2) as a critical factor, and, indeed, ectopic expression of ACE2 increased susceptibility of gastric organoid-derived monolayers to SARS-CoV-2. ACE2 expression pattern in GI biopsies of patients mirrors SARS-CoV-2 infection levels in monolayers. Thus, local ACE2 expression limits SARS-CoV-2 expression in the GI tract to the intestine, suggesting that the intestine, but not the stomach, is likely to be important in viral replication and possibly transmission.

## Highlights

• SARS-CoV-2 robustly infects intestinal, but not gastric, organoid-derived monolayers
• The infection tropism is matched by ACE2 expression in organoid-derived monolayers
• Ectopic expression of ACE2 indicates that ACE2 is the limiting factor
• Histology confirms the ACE2 expression pattern also in patient tissues

## Graphical abstract

**Figure:**
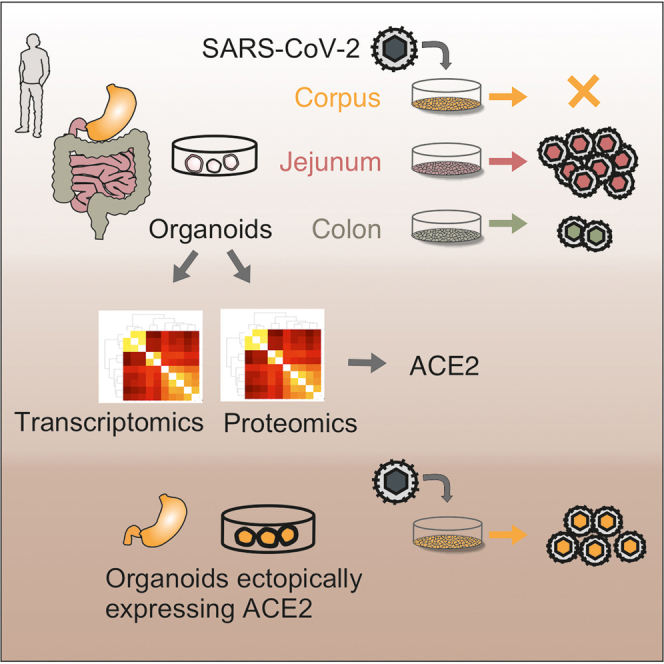

## Teaser

Bartfeld and colleagues examined the susceptibility of gastrointestinal organoid-derived monolayers to SARS-CoV-2 infection. They observed productive infection in intestinal, but not gastric, cells. Susceptibility correlated with ACE2 expression in organoids and patient tissues. Ectopic ACE2 expression rendered gastric organoid-derived monolayers susceptible to SARS-CoV-2, leading to the conclusion that ACE2 expression in gastrointestinal epithelium is the key limiting factor for SARS-CoV-2 infection.

## Introduction

Over the last two decades, several viruses of zoonotic origin from the *Coronaviridae* family have become a global health concern. Severe acute respiratory syndrome-related coronavirus (SARS-CoV) and Middle East respiratory syndrome coronavirus (MERS-CoV) caused outbreaks of severe respiratory diseases, and severe acute respiratory syndrome coronavirus 2 (SARS-CoV-2) caused the global pandemic of COVID-19 (Zhu et al., 2020).

Severity of COVID-19 is highly variable and ranges from asymptomatic infections to severe respiratory system failure and multi-organ failure. Symptoms mainly concern the respiratory tract yet can also include diarrhea and vomiting, pointing to an involvement of the gastrointestinal (GI) tract (Neurath, 2020). On the molecular level, sequencing and histology data showed that the SARS-CoV-2 entry receptor, angiotensin-converting enzyme 2 (ACE2), is highly expressed in the GI tract at RNA and protein levels (Hikmet et al., 2020; Qi et al., 2020).

Organoids, 3-dimensional primary cell cultures derived from stem cells, and organoid-derived monolayers have been used to study SARS-CoV-2 infection and cell-type tropism. In these studies, SARS-CoV-2 robustly infects small intestinal cells and colonic cells, and to a certain extent also pediatric and fetal gastric cells (Zhou et al., 2020; Lamers et al., 2020; Stanifer et al., 2020; Giobbe et al., 2021; Heuberger et al., 2021). Immunofluorescence imaging pointed to SARS-CoV-2 tropism to enterocytes (Lamers et al., 2020; Zhou et al., 2020). In fetal and pediatric gastric organoids, SARS-CoV-2 showed tropism to somatostatin-positive enteroendocrine cells (Giobbe et al., 2021).

While a general involvement of the GI tract in SARS-CoV-2 infection is clear, most of the studies focused on individual segments of GI tract such as stomach, ileum, or colon, and there is a lack of direct side-by-side comparisons of SARS-CoV-2 entry factors and viral infection in the major GI tract segments.

Here, using adult stem cell-derived organoids and their derived monolayers, we examine SARS-CoV-2 infection in the gastric (corpus), small intestinal, and colonic epithelium. While small intestinal epithelium and colonic epithelium were susceptible, the gastric epithelium was protected from infection. This was mirrored in the expression of ACE2. Ectopic expression of ACE2 in corpus monolayers increased the susceptibility to SARS-CoV-2 infection. Our results indicate that SARS-CoV-2 tropism in GI tract is limited by localized expression of ACE2 in the GI tract.

## Results

### SARS-CoV-2 shows differential infection tropism in GI organoid-derived monolayers

**Figure 1:**
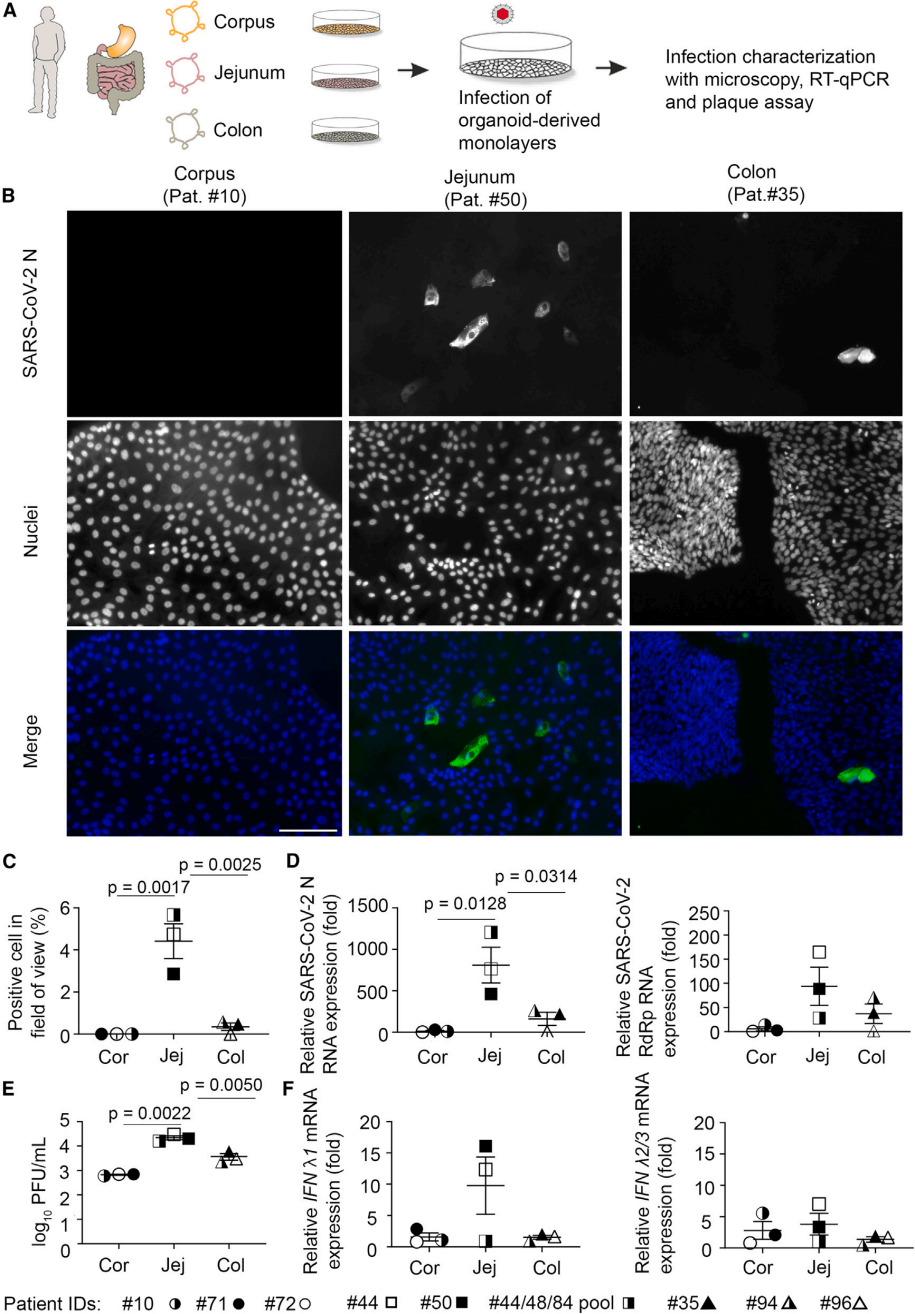
SARS-CoV-2 infects small intestinal and colonic, but not gastric, organoid-derived monolayers (A) Scheme of experimental setup. Organoid-derived monolayers were differentiated for 4 days before incubation with 1 × 10^6^ PFU of SARS-CoV-2. The infection was characterized 24 hpi. (B) SARS-CoV-2 nucleocapsid (N) protein (green) stained by immunofluorescence. Nuclei were stained with Hoechst 33342. Scale bar, 100 μm. (C) Quantification of the staining shown in (B). Ten images were quantified per donor. (D) SARS-CoV-2 N and RdRp RNA quantification using RT-qPCR in monolayer lysates. Data were normalized to *18S* ribosomal RNA. (E) Quantification of infectious virus particles in the supernatant of infected monolayers using plaque assay. (F) Type III interferon (IFNλ) *IFNλ1* and *IFNλ2/3* RNA quantification in infected monolayer lysates. Data in (C)–(F) presented as mean of 3 individual donors or pools as indicated ±SEM. Statistical analysis was carried out using one-way ANOVA with Tukey’s multiple comparisons test.

To compare the infection in the three major GI segments stomach, small intestine, and colon, we focused on the large regions corpus, jejunum, and colon. Gastric, jejunal, and colonic organoids were expanded and seeded into monolayers, and monolayers were differentiated for 4 days. Differentiated monolayers expressed regional markers as shown before (Stanifer et al., 2020; Aguilar et al., 2022; Figure S1). Monolayers were infected with 1 x 10^6^ plaque-forming units (PFUs) of SARS-CoV-2 and examined 24 h post-infection (hpi) (Figure 1A). Immunofluorescence staining and RT-qPCR for SARS-CoV-2 nucleocapsid (N) protein and RT-qPCR for viral RNA-dependent polymerase (RdRp) indicated that jejunal cells were most susceptible to SARS-CoV-2 infection; colonic cells were also susceptible, but infection was not detected in gastric cells (Figures 1B–1D). Plaque assay, which measures infectious virus in the supernatant of infected cells, showed significantly higher number of viral particles (2.5 × 10^4^ PFU/mL) in jejunal than colonic monolayers (3.75 × 10^3^ PFU/mL) and corpus monolayers (6.333 × 10^2^ PFU/mL) (Figure 1E), indicating most productive infection in the jejunum. Host innate type III interferon response was most pronounced in jejunal monolayers (Figure 1F). Together, this indicated a preferential tropism of SARS-CoV-2 for small intestinal cells.

### ACE2 is differentially expressed between GI segments

**Figure 2:**
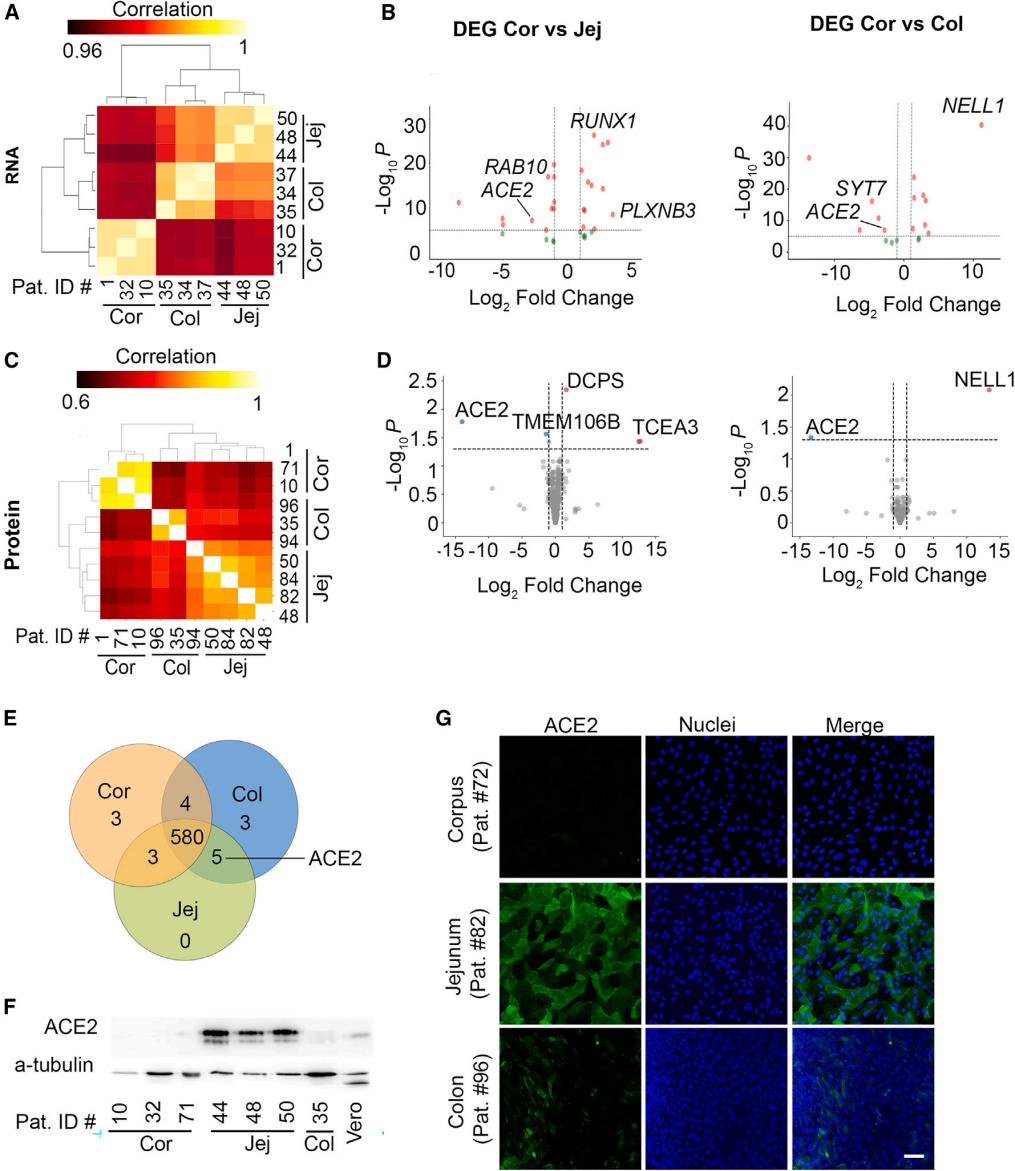
ACE2 is not expressed in corpus organoids and organoid-derived monolayers (A) Published data were re-analyzed (Kayisoglu et al., 2021) to show hierarchical clustering of total RNA transcriptome of corpus, jejunum, and colon organoids. (B) Volcano plot visualizing genes associated with SARS-CoV-2 infection by published CRISPR screens and differentially expressed in corpus and jejunum (left) or corpus and colon (right) organoids. Red dots visualize DEG with *p* ≤ 0.05 and ≥2 log2 fold change. Green dots visualize DEG with *p* ≥ 0.05 and ≤2 log2 fold change. See Table S2. (C) Hierarchical clustering of corpus, jejunum, and colon 3D organoids total proteome. (D) Volcano plot visualizing proteins associated with SARS-CoV-2 infection by CRISPR screens and differentially expressed in corpus and jejunum (left) or corpus and colon (right) organoids. Blue dots visualize proteins downregulated with *p* ≤ 0.05 and ≤-1 log2 fold change. Red dots identify upregulated proteins with *p* ≤ 0.05 and ≥1 log2 fold change. See Table S3. (E) Venn diagram of host proteins associated with SARS-CoV-2 infection. See Table S4. (F) ACE2 expression analysis using western blot in organoids and Vero cell line as control. (G) Immunostaining of ACE2 (green) in differentiated organoid-derived monolayers. Nuclei stained with Hoechst 33342. Scale bar is 30 μm.

To understand the mechanism underlying the difference in infection in the GI segments, we used our existing dataset of RNA expression in organoids (Kayisoglu et al., 2021) and focused on the 3 major regions corpus, jejunum, and colon. RNA expression profiles of the 3 GI segments clustered together (Figure 2A). Region-specific markers were expressed as expected (Figure S2). To search for host factors that may influence SARS-CoV-2 infection in the GI tract, we generated a list of host factors identified by CRISPR screens (Table S1). Common genes in the list of host factors and differentially expressed genes (DEGs) between stomach and both intestinal segments, in our dataset, included *ACE2* (Figure 2B; Table S2). To verify these data on protein level, we performed proteome analysis of the organoids from the corpus, jejunum, and colon. Protein expression profiles of organoids clustered together according to the GI segment they were derived from (Figure 2C). Differential expression analysis of the proteome using the same list of host factors as for RNA sequencing analysis highlighted ACE2 among other proteins (Figure 2D), and ACE2 was one of only five proteins that were expressed commonly in the jejunum and colon, but not in the corpus (Figure 2E; Tables S3 and S4). Western blot of ACE2 confirmed the relative abundance of ACE2 in intestinal organoids compared to no detectable protein in corpus organoids (Figure 2F). Immunofluorescence staining of ACE2 also showed that the protein is not detectable in gastric organoid-derived monolayers (Figure 2G). Additional SARS-CoV-2 entry factors transmembrane serine protease 2 (*TMPRSS2*), Neuropilin 1 (*NRP1*), and transmembrane protein 106B (*TMEM106B*) were expressed in the gastric organoids and organoid-derived monolayers and thus are unlikely to be the limiting factor (Figure S3). We concluded that ACE2 is not expressed in gastric corpus organoids or organoid-derived monolayers. We hypothesized that the lack of this main entry receptor for SARS-CoV-2 may underlie the protection of the gastric organoid-derived monolayers from infection with this virus.

### Lentiviral ACE2 expression in corpus organoids increases susceptibility to SARS-CoV-2 infection

**Figure 3:**
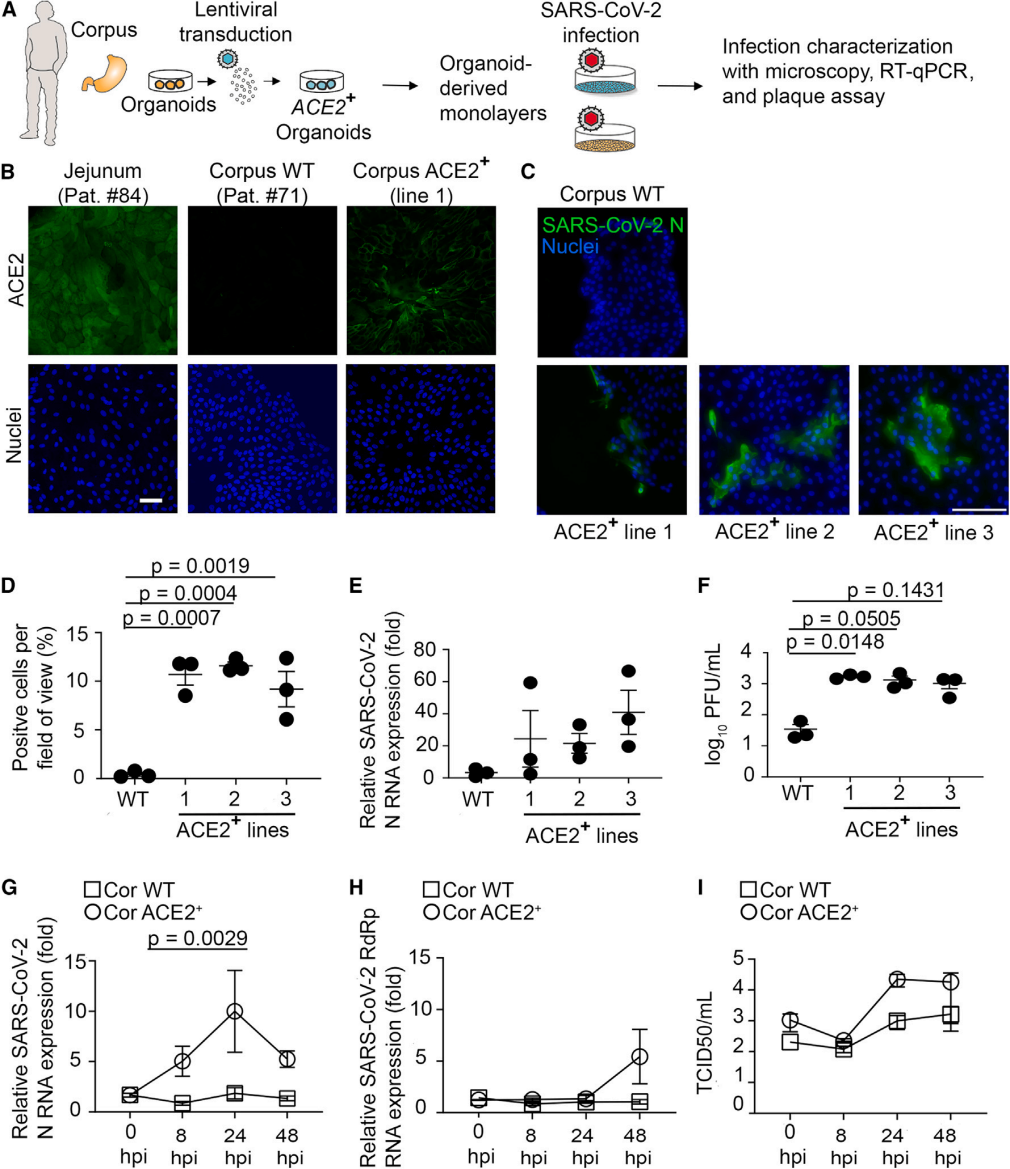
Ectopic ACE2 expression increases the susceptibility of differentiated corpus monolayers for SARS-CoV-2 infection (A) Scheme of experimental setup. Parental line (wild-type [WT]) and ectopically ACE2-expressing corpus (ACE2^+^ corpus) lines are compared. Organoid-derived monolayers were differentiated for 4 days before infection with 1 × 10^6^ PFU of SARS-CoV-2. Infection was characterized at 24 hpi. (B) Immunostaining of ACE2 expression. Scale bar is 30 μm. (C) Immunostaining of SARS-CoV-2 N protein. Scale bar is 100 μm. (D) Quantification of immunofluorescence shown in C. Ten images were quantified per line. (E) Quantification of SARS-CoV-2 N RNA by RT-qPCR normalized to *GAPDH*. (F) Quantification of infectious virus particles in the supernatant by plaque assay. (G) SARS-CoV-2 N RNA quantification at different time points after infection using RT-qPCR. Data were normalized to *GAPDH*. (H) SARS-CoV-2 RdRp RNA quantification at different time points after infection. Data were normalized to *GAPDH*. (I) Quantification of infectious virus production over time by TCID_50_ assay. Data in (D), (E), and (F) presented as mean ± SEM of 3 independent experiments. Data in (G), (H), and (I) presented as mean ± SEM of 3 organoid lines. Statistical analysis was carried out using one-way ANOVA with Tukey’s multiple comparisons test.

To analyze, whether ACE2 expression is the limiting factor for SARS-CoV-2 infection in corpus monolayers, we generated corpus organoid lines ectopically expressing ACE2 (ACE2^+^ corpus) (Figure 3A). Expression of ACE2 in the transduced cells was well observable by immunofluorescence staining, albeit still lower than ACE2 expression in jejunal cells (Figure 3B). Three ACE2-expressing lines of corpus organoids of one patient (#71) were established and infected with SARS-CoV-2 as earlier. Immunofluorescence microscopy for SARS-CoV-2 nucleocapsid protein (N) indicated that ACE2^+^ corpus organoid-derived monolayers were susceptible to SARS-CoV-2 infections (Figure 3C) with infection rate of 9.6%–11.6% (Figure 3D). Quantification of SARS-CoV-2 nucleocapsid (N) RNA by RT-qPCR confirmed this susceptibility (Figure 3E). Plaque assay showed significantly higher number of infectious virus particles in the supernatant of ACE2^+^ corpus cells, indicating productive virus replication (Figure 3F), yet the assay also showed some infectious virus in the supernatant of the wild-type (WT) corpus cells, as was already observed in Figure 1E. We suspected that these were residual virus particles from the initial high infection dose that generally escape washing steps. To address this point, we examined SARS-CoV-2 growth in ACE2^+^ corpus organoid-derived monolayers over time. The viral N RNA started to increase 8 hpi and peaked at 24 hpi. The later time point of 48 hpi showed an increase in SARS-CoV-2 RdRp RNA levels (Figures 3G and 3H). Using tissue culture infection dose (TCID)_50_ assay we observed increased virus release at 24 hpi, which remained stable until 48 hpi (Figure 3I).

In conclusion, ectopic ACE2 expression in corpus organoid-derived monolayers resulted in increased susceptibility to SARS-CoV-2 infection, indicating that the absence of ACE2 is the central factor preventing infection of human gastric epithelium.

### ACE2 expression in GI tissue mirrors expression in organoids

**Figure 4:**
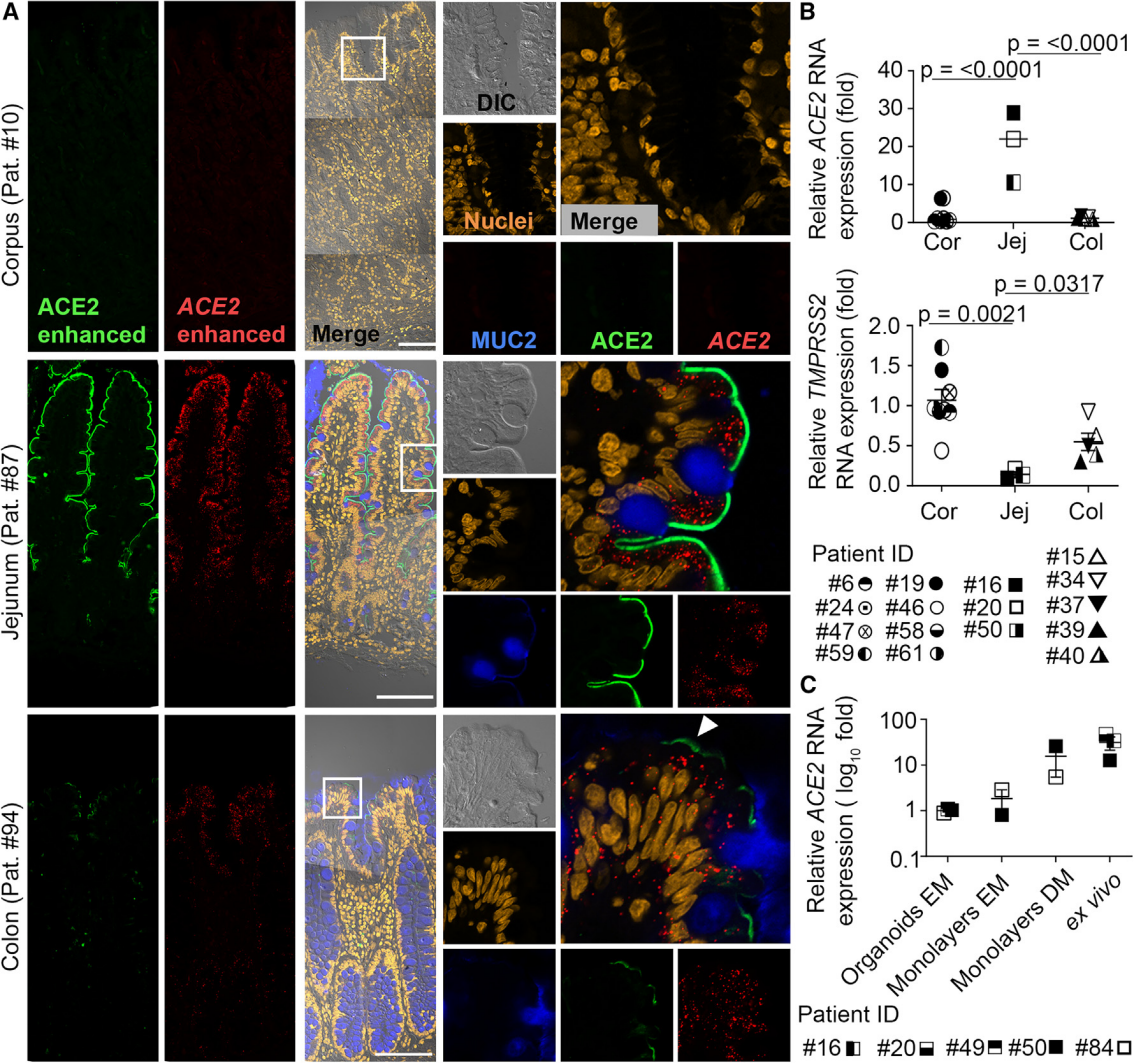
Differential expression of ACE2 in gastrointestinal tissue (A) Immunostaining of MUC2 protein (blue), ACE2 protein (green), and *in situ* hybridization of *ACE2* mRNA (red) in tissue biopsies. Left column with enhanced green and red intensity to visualize expression in colon. Nuclei were stained with DAPI (yellow). Scale bar is 30 μm. Images are representative of tissues from 2 patients per GI segment. (B) Relative mRNA levels of *ACE2* (upper panel) and *TMPRSS2* (lower panel) in total RNA from tissue biopsies using RT-qPCR. Data were normalized to *GAPDH* and compared to corpus mRNA levels. Data presented as mean ± SEM of indicated individual donors per GI segment. Statistical analysis was carried out using one-way ANOVA with Tukey’s multiple comparisons test. (C) Relative mRNA levels of *ACE2* in RNA isolations from organoids, organoid-derived monolayers in expansion medium (EM), differentiated monolayers in differentiation medium (DM), and *ex vivo*-isolated epithelium using RT-qPCR. Data were normalized to *GAPDH* and compared to organoid mRNA levels. Data presented as mean ± SEM of individual donors.

To verify the expression pattern of ACE2 in human tissue, we used RNAscope and immunofluorescence staining in GI tissue biopsies and RT-qPCR of *ex vivo*-isolated epithelium crypts. ACE2 was not detectable at protein or RNA level in gastric glands. The highest ACE2 expression and abundance of *ACE2* mRNA were observed in jejunal sections, particularly the villus regions (Figure 4A). In the colon, ACE2 expression was restricted to a few cells and at lower intensities as compared to the jejunum. In some cells, the *ACE2* RNA signal does not fully correspond to protein expression. MUC2, which marks intestinal goblet cells, was used as a control staining, and, while negative in the corpus, as expected, it also showed that ACE2 was absent from MUC2-positive goblet cells. In the colon, where goblet cells are highly abundant, only few MUC2-negative cells expressed apical ACE2 protein (Figure 4A, arrowhead). Analysis of published datasets from single-cell RNA sequencing of human small and large intestine shows that the *ACE2*-expressing population overlaps with populations expressing markers of enterocytes (Figure S4). RT-qPCR of tissue-isolated total RNA showed again the highest *ACE2* mRNA levels in jejunum tissue, compared to mRNA levels in colon or corpus tissue, but it did not pick up a difference between the corpus and colon (Figure 4B). As control, we also examined *TMPRSS2* expression, which was expressed throughout the GI tract as measured by RT-qPCR (Figure 4B). These matched the expression patterns identified in transcriptomic and proteomic data analysis (Figure S3). To allow a direct comparison of organoids, their derived monolayers, and differentiated monolayers and tissue, we probed *ACE2* using RT-qPCR in jejunum samples. We observed that differentiation of monolayers strongly increased *ACE2* expression (Figure 4C), matching to the observation that crypts did not show *ACE2* mRNA in the in situ hybridisation (ISH).

Together, the data indicated that the expression of ACE2 that we observed in organoids and organoid-derived monolayers was mirroring the expression in the tissue: the corpus was devoid of ACE2, the jejunum expresses high levels of ACE2, especially the non-secretory cells of the villus, and the colon expresses lower levels of ACE2. We conclude that the infection of SARS-CoV-2 is restricted by the presence of ACE2 on GI epithelial cells.

## Discussion

Clinical reports have pointed to a GI component in COVID-19 disease: approximately 10%–30% of patients present with diarrhea (Neurath, 2020), and the virus is detected in stool samples (Zhang et al., 2020; Zhou et al., 2020). In the present study, we performed a side-by-side comparison of organoid-derived monolayers from the three major segments of the GI tract. We find that the virus infection is strongest in jejunal and not detectable in gastric epithelial cells. We identify the virus entry receptor ACE2 as the limiting factor for infection in gastric cells.

The importance of GI infection in COVID-19 is not yet understood. A large multicenter study reported that GI symptoms correlate with lower severity of COVID-19 (Livanos et al., 2021), but specific populations may also be correlated with higher severity of COVID-19 (Jin et al., 2020). Thus, depending on the study, it was speculated that GI symptoms may attenuate inflammatory response to SARS-CoV-2, thus leading to a milder disease (Livanos et al., 2021), or that GI symptoms may lead to higher electrolyte disturbances, aggravating disease (Neurath, 2020). Therefore, the importance of GI infection in the course of the disease remains unclear and deserves further investigation.

The data presented here corroborate findings that SARS-CoV-2 can robustly infect small intestinal and colon epithelium. Similarly to published studies, we observed production of viral RNA and viral protein in infected cells (Lamers et al., 2020; Zang et al., 2020; Beumer et al., 2021; Zhao et al., 2021). In our data, compared to jejunum, colon organoid-derived monolayers were less susceptible to infection, which correlated with lower ACE2 expression levels. The staining in tissue sections demonstrate that, in the colon, ACE2 is only expressed by a subpopulation of cells, which are not goblet cells. Available single-cell transcriptomic data indicate that absorptive enterocytes are expressing the highest levels of *ACE2* (Figure S4). One other report has compared 12 lines of terminal ileum and 13 lines of ascending colon and found very high variation of ACE2 expression between the individual lines, but no statistically significant difference between the ileum and colon (Jang et al., 2022). The use of different expansion media, either promoting expansion of undifferentiated cells (Jang et al., 2022) or promoting also expansion of secretory cells (Fujii et al., 2018), which we use here, influences the presence of goblet cells and thus likely also susceptibility to SARS-CoV-2 infection. The comparison with patient tissue suggests that indeed ACE2-expressing cells are much less prominent in the colon than in the jejunum.

The data presented here show no expression of ACE2 in the stomach and in gastric organoids. During the early stages of the SARS-CoV-2 pandemic, single case reports showed SARS-CoV-2 infection in gastric tissue as well as in lower regions of the GI tract (Xiao et al., 2020). However, single-cell RNA sequencing data and patient biopsy data later suggested that ACE2 expression in the esophagus and stomach is related to the development of intestinal metaplasia in gastric tissue (Xu et al., 2020; Jin et al., 2021), indicating that healthy gastric epithelium does not express ACE2. The ectopic expression of ACE2 in the corpus, which we perform here, could mimic the situation in patients’ pathological conditions such as intestinal metaplasia in gastric or esophagus epithelium. These regions of high ACE2 expression allow SARS-CoV-2 infection as suggested by others (Xu et al., 2020; Jin et al., 2021).

A previous study has focused on fetal and pediatric gastric epithelium and demonstrated some susceptibility to SARS-CoV-2 infection and ACE2 expression in organoids derived from these tissues (Giobbe et al., 2021). In comparison to the fetal and pediatric organoids, adult organoids were poorly infectible, and the classical plaque assay could not detect virus release from the infected cells. It is possible that ACE2 expression changes with age in the GI tract epithelium, as is suggested by higher abundance of ACE2 reads in pediatric or fetal organoids compared to adult organoids in the RNA sequencing data (Giobbe et al., 2021).

Our data underline that SARS-CoV-2 can infect cells within the intestine, but not the stomach. It is likely that the small intestine is the prime site of viral replication.

## Experimental procedures

### Resource availability

#### Lead contact

Further information and requests for resources and reagents should be directed to and will be fulfilled by the corresponding author, Sina Bartfeld (s.bartfeld@tu-berlin.de).

#### Materials availability

There are restrictions to the availability of human organoid cultures due to the limitations of the consent that was given by the patients and the guidance of the ethical committee.

#### Data and code availability

The mass spectroscopy proteomics data were deposited to PRlDE with the data identifier PXD044789.

### Organoid culture and 2D monolayer seeding

GI tissue biopsies were obtained from donors that underwent surgical resection at the University Hospital of Würzburg (see supplemental experimental procedures). The acquisition of patient material was approved by the ethical committee of the University of Würzburg (Approval 37/16), and informed consent was obtained from all donors. Organoids were generated following previously published protocols (Fujii et al., 2018; Kayisoglu et al., 2021) (see supplemental experimental procedures).

Monolayers were generated from organoids 7 days after seeding. Organoids were collected in Advanced DMEM (AD++), centrifuged at 450 g for 5 min, resuspended in TrypLE Express (Gibco), and mechanically disrupted followed by incubation at 37°C for 10 min. Single-cell suspension was washed and centrifuged at 450 g for 5 min. Single cells were seeded in 48-well plate (833923, Sarstedt) using the expansion media supplemented with Rho kinase inhibitor (RHOKi, 10 μM, Y-27632, Sigma-Aldrich). The following day, expansion media was replaced with differentiation media without Wnt for all GI segments. For intestinal segments, FGF-2, IGF-1, 50% R-Spondin, and 50% Noggin were removed (Stanifer et al., 2020; Aguilar et al., 2022) (see supplemental experimental procedures). Monolayers were grown for 4 days under differentiation condition before the SARS-CoV-2 infection.

Ectopic expression of ACE2 was performed as published before (Hönzke et al., 2022).

## Supplemental information

Document S1. Supplemental experimental procedures, Figures S1–S4, and Tables S3 and S4

Table S1. Host factors identified by published CRISPR screens, related to Figure 2B

Table S2. Differentially expressed genes between corpus and jejunum or corpus and colon, related to Figure 2B

Document S2. Article plus supplemental information
