# Supplementary material for: SARS-CoV-2 tropism to intestinal but not gastric epithelial cells is defined by limited ACE2 expression
Source: Stem Cell Reports. 2024 Apr 25;19(5):629–38. doi: 10.1016/j.stemcr.2024.03.008 (PMC11103887; doi:10.1016/j.stemcr.2024.03.008)
Supplement: Document S1. Supplemental experimental procedures, Figures S1–S4, and Tables S3 and S4 [file mmc1.pdf]

Stem Cell Reports, Volume *19*

Supplemental Information

SARS-CoV-2 tropism to intestinal but not gastric epithelial cells is defined by limited ACE2 expression

Mindaugas Paužuolis, Diana Fatykhova, Boris Zühlke, Torsten Schwecke, Mastura Neyazi, Pilar Samperio-Ventayol, Carmen Aguilar, Nicolas Schlegel, Simon Dökel, Markus Ralser, Andreas Hocke, Christine Krempl, and Sina Bartfeld

Supplementary Material

- **Supplementary Figures**
- **Supplementary Tables and Legends**
- **Supplementary Experimental Procedures**
- **Supplementary References**

Supplementary Figures

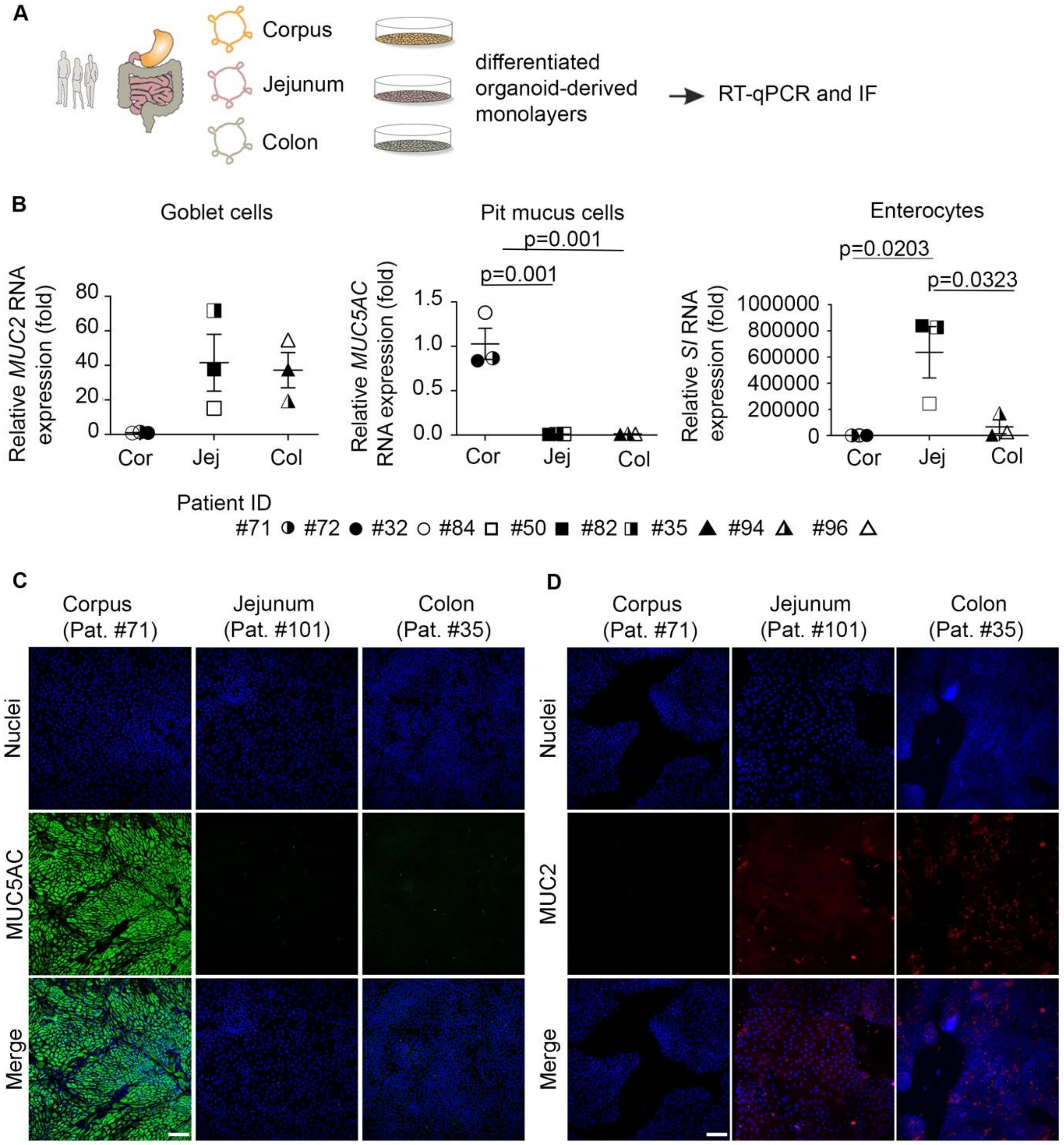

Figure S1: Differentiated GI organoid-derived monolayers express markers of differentiated cells.

A. Schematic setup.

B. Relative mRNA levels of enterocyte (sucrase-isomaltase (*SI*)), goblet cell (*MUC2*), gastric pit cell (*MUC5AC*) markers in RNA isolated from organoid-derived monolayers using RT-qPCR. Data were normalized to *GAPDH* and compared to corpus mRNA levels. Data presented as mean of 3 individual donors ± SEM. Statistical analysis was performed using one-way ANOVA test with Tukey's multiple comparisons test.

C - D. Immunostaining of MUC5AC (green) and MUC2 (red) in differentiated corpus, jejunum and colon organoid-derived monolayers respectively. Nuclei stained with Hoechst 33342. Scale bar is 30 µm.

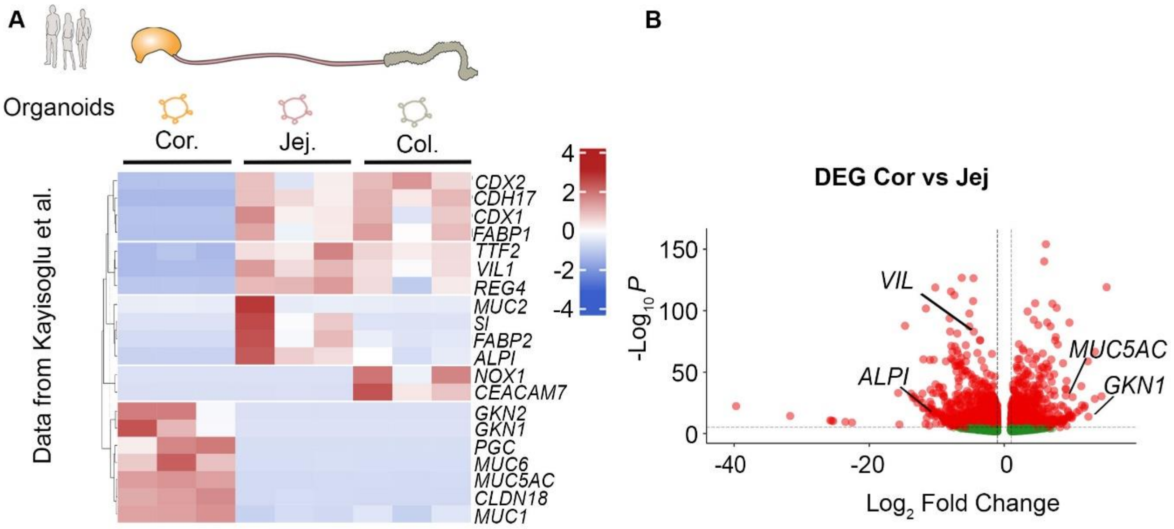

Figure S2: GI organoids show segment-specific expression of GI cell markers.
A. Normalized and scaled expression of selected gastric and intestinal cell markers in corpus, jejunum and colon organoids in GI organoid RNA sequencing data[1].
B. Volcano plot of differentially expressed genes between corpus and jejunum in transcriptomic data[1].

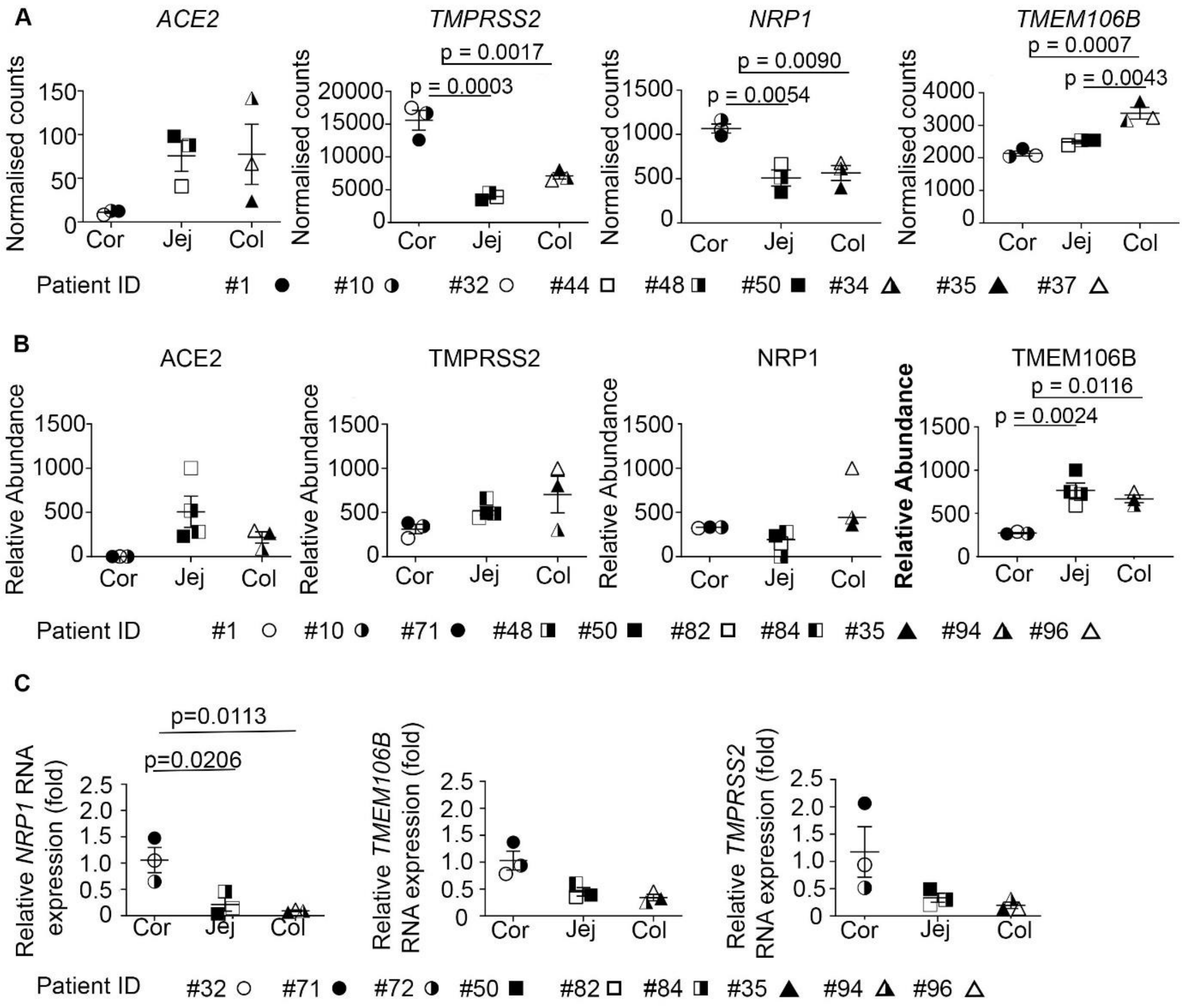

Figure S3: GI expression of SARS-CoV-2 entry factors ACE2, TMPRSS2, NRP1 and TMEM106B in organoids and organoid-derived monolayers.

A. *ACE2, TMPRSS2, NRP1*[2] and *TMEM106B* [3] RNA expression in corpus (Cor), jejunum (Jej), and colon (Col) organoids from published data (Kayisoglu et al. 2021). Data presented as mean ± SEM of 3 individual donors per GI tract segment. Statistical analysis was performed using one-way ANOVA test with Tukey's multiple comparisons test.

B. ACE2, TMPRSS2, NRP1 and TMEM106B protein relative abundance in corpus (Cor), jejunum (Jej) and colon (Col) organoids. Data presented as mean± SEM of 3 individual donors per GI tract segment. Statistical analysis was performed using one-way ANOVA test with Tukey's multiple comparisons test.

C. Relative mRNA levels of *NRP1*, *TMEM106B* and *TMPRSS2* in RNA isolated from differentiated gastrointestinal organoid-derived monolayers quantified using RT-qPCR. Data were normalized to *GAPDH* and compared to average of corpus mRNA levels. Data presented as mean± SEM of 3 individual donors per GI segment. Statistical analysis was performed using one-way ANOVA test with Tukey's multiple comparisons test.

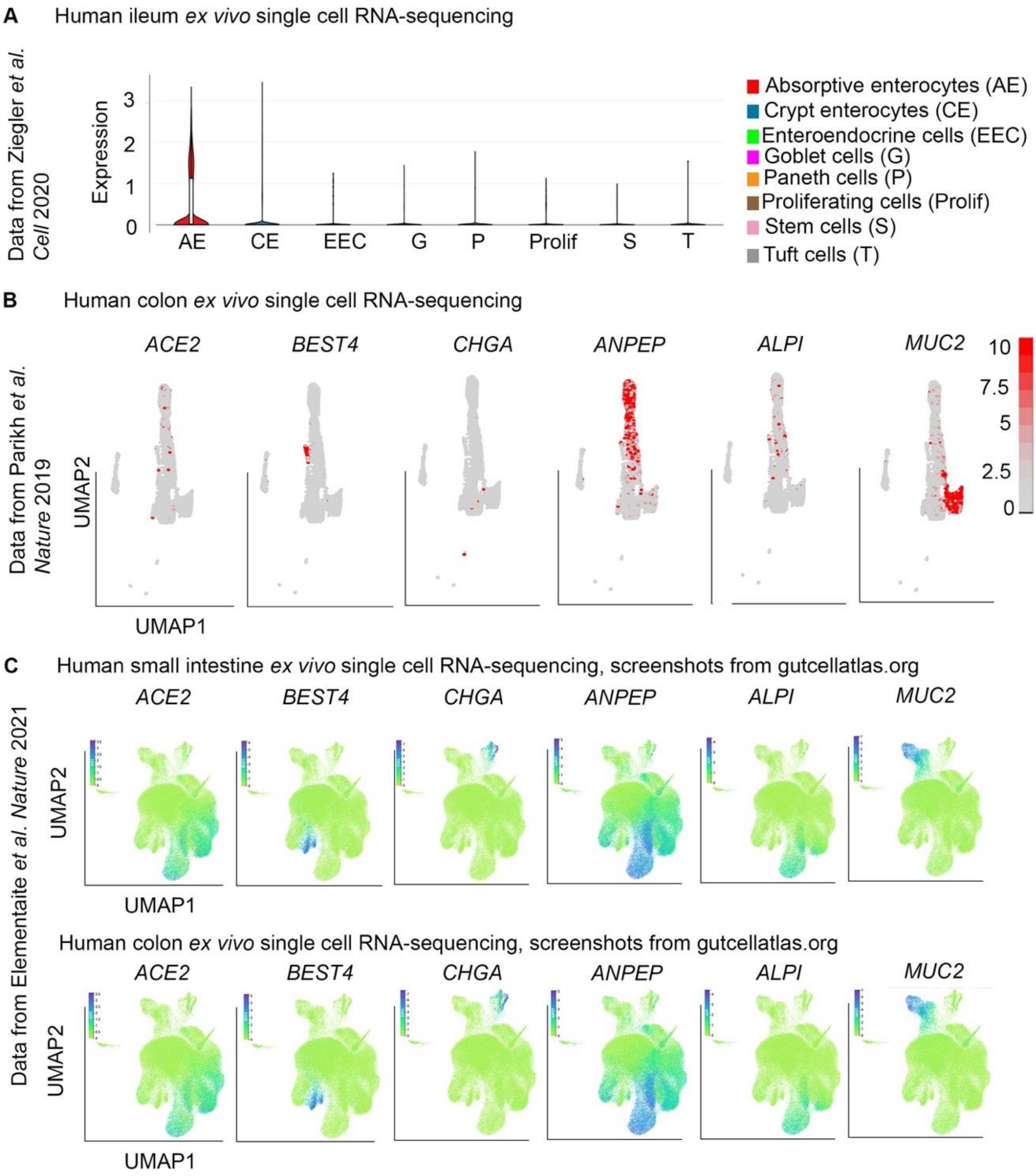

Figure S4: *ACE2* expressing cells overlap with populations expressing markers of enterocytes. A. *ACE2* expression in different cellular populations from human *ex vivo* ileum tissue[4]. Data were visualized using Broad Institute single cell portal (singlecell.broadinstitute.org). The highest expression of *ACE2* is in the subpopulation of absorptive enterocytes, but not enteroendocrine cells.
B. Re-analysis of the human *ex vivo* colon tissue data[5] shows expression of *ACE2* in populations that express *ANPEP* and *ALPI*, but not *BEST4*, *CHGA,* or *MUC2*.
C. The expression of *ACE2* co-localized in populations that express *ANPEP* and *ALPI*, but not *BEST4*, *CHGA,* or *MUC2* in human *ex vivo* intestinal and colon tissue[6] . Data were visualized using Gut Cell Atlas (gutcellatlas.org). Specific option selection in gutcellatlas.org: 1) age group: adult. 2) category: Epithelial 3) Region: LargeInt or SmallInt.

Supplementary Tables and Legends

Table S1. Host factors identified by published CRISPR screens. Related to Figure 2B.

See separate spreadsheet.

Table S2. Differentially expressed genes between corpus and jejunum or corpus and colon. Related to Figure 2B.

See separate spreadsheet.

Table S3. Significantly differentially expressed proteins between corpus and jejunum or corpus and colon 3D organoids. Related to Figure 2D.

Protein	Corpus mean	Jejunum mean	Colon mean	p Welch	T-test ind	p Bartlett	pval corrected	Fold change	log2 fold change
DCPS	18,25707	16,63789		8,59E-06	8,59E-06	0,970697	0,004519	3,072006	1,619181
ACE2	0	13,98436		6,27E-05	6,27E-05	0	0,016484	6,17E-05	-13,9844
TMEM106B	14,82227	16,21215		0,000155	0,000155	0,258588	0,027248	0,381598	-1,38987
NELL1	13,34358	0,653031		0,000277	0,000277	0,009806	0,036438	6610,526	12,69055
TCEA3	13,25163	0,78449		0,000351	0,000351	0,060611	0,036921	5662,174	12,46714
NDUFB3	17,79708	18,76953		0,000429	0,000429	0,49187	0,037638	0,509638	-0,97245
NELL1	13,34358		0	1,58E-05	1,58E-05	0	0,008319	10394,84	13,34358
ACE2	0		13,31917	0,000176	0,000176	0	0,046216	9,78E-05	-13,3192

Explanation of column headings:
P Welch: P value of Welch test,
T-test ind: T-test independent sample.
P Bartlett: p value of Bartlett test.
P val corrected: p value corrected using Benjamini-Hochberg test for multiple testing.
Fold change: protein fold change.
log2 fold change: log2 fold protein change.

Table S4. Unique and shared host proteins associated with SARS-CoV-2 infection detected in corpus, jejunum and colon organoid proteome. Related to Figure 2E.

Unique in corpus	Unique in jejunum	Unique in colon	Corpus and colon	Corpus and jejunum	Jejunum and colon
NCAPH2		PRMT7	POLK	NELL1	ACE2
EGR1		HOXB9	TCEA3	PLXNB3	ARPP19
LSM11		CENPW	WEE1	SLC35B4	BRI3
			ZBTB14		CARD10
					SLC25A26

Supplementary Experimental Procedures

List 1: GI tissue donor data.

GI organoid	Patient ID	Age	Gender	Surgical procedure	Carcinoma
Corpus	1	32	F	Sleeve gastrectomy	No
	6	29	F	Sleeve gastrectomy	No
	10	37	F	Sleeve gastrectomy	No
	19	45	M	Sleeve gastrectomy	No
	24	29	F	Sleeve gastrectomy	No
	32	47	F	Sleeve gastrectomy	No
	46	54	F	Gastric carcinoma resection	Yes
	47	75	M	Gastric carcinoma resection	Yes
	58	58	F	Gastric carcinoma resection	Yes
	59	31	M	Sleeve gastrectomy	No
	61	71	F	Gastric carcinoma resection	Yes
	71	82	F	Gastric carcinoma resection	Yes
	72	79	M	Gastric carcinoma resection	Yes
Jejunum	16	-	-	-	-
	20	43	F	Stomach bypass	No
	44	34	F	Stomach bypass	No
	49	64	F	Stomach bypass	No
	50	53	M	Stomach bypass	No
	48	50	M	Stomach bypass	No
	82	41	F	Stomach bypass	No
	84	44	F	Stomach bypass	No
	87	52	F	-	-
	101	32	M	Whipple operation	No
Colon	15	87	M	-	-
	34	52	M	Sigmoid carcinoma resection	Yes
	35	76	F	Hemicolectomy	Yes
	37	74	F	Colon carcinoma resection	Yes
	39	68	F	Coecum carcinoma resection	Yes
	40	66	M	-	-
	94	62	F	Sigmoid resection	No
	96	79	M	Colon carcinoma resection	Yes

SARS-CoV-2 infection

Gastrointestinal monolayers were inoculated with 50 µl of SARS-CoV-2[7], containing $1x10^6$ plaque-forming units per well, and incubated for 1h (37°C, 5% CO2) with periodical shaking. After incubation, the inoculum was removed, and monolayers were washed 3 times with PBS. Monolayers were incubated in differentiation media for 24 h before collection.

SARS-CoV-2 N protein IF staining in gastrointestinal monolayers

SARS-CoV-2 protein expression was evaluated with SARS N protein staining infected monolayers. After infection, cells were fixed with 4 % paraformaldehyde for 30 min at RT, washed with PBS two times, and permeabilized with 0.5 % Triton X-100 in PBS for 15 min. Mouse anti-SARS-CoV N (1:1000, MM05, Sino Biologicals) was diluted in PBS and incubated at RT for 1h. The secondary antibody (1:500 goat anti-mouse Alexa Fluor-488, A-11001, Thermo Fisher Scientific) was diluted in PBS and incubated at RT for 1h. Nuclei were stained with Hoechst 33342 (1:5,000; H3570, Life Technologies). Images of infected cells were acquired with EVOS™ FL Digital Inverted Fluorescence Microscope (Invitrogen).

SARS-CoV-2 RNA isolation and viral titer quantification using RT-qPCR

Gastrointestinal monolayers were lysed using TRI Reagent and RNA was isolated following the protocol of Direct-zol RNA MiniPrep (R2052, Zymo Research). cDNA was generated using random

primers (48190011, Life Technologies) and M-MuLV reverse transcriptase (M0253, New England Biolabs) according to the manufacturer's protocol. Quantitative reverse transcription PCR was carried out using SYBR Green Supermix (172-5270, BioRad) in a CFX96 Touch Real-Time PCR detection system using Bio-Rad CFX Manager software (BioRad). Viral titers were normalized to non-infected control using the $\Delta\Delta$Ct values method and normalized to the *18S* gene. List of all primers used in the study are listed below in the section RT-qPCR.

Plaque assay

Plaque assay was carried out using Vero TMPRSS2 cells. Cells were cultured in DMEM w/Glutamax (12634028, Thermo Fisher) supplemented with 10% FCS (S0615, Biochrom) and 1% P/S (15140122, Thermo Fisher). The supernatant of infected cells was serially diluted before the inoculation of Vero TMPRSS2 cells. Inoculated cells were incubated at 37°C for 1 hour with periodical shaking. The cells were overlayed with 1ml of 0.6 % methylcellulose overlay and incubated for 4 days at 37°C and 5% CO_2. Plaques were fixed with 20% Methanol solution supplemented with 0.2 % Crystal violet and incubated overnight. The total number of plaques per well was counted and used to calculate PFU/ml.

GI organoid culture

Organoids were cultured in Advanced DMEM/F12 supplemented with GlutaMAX, 10 mM HEPES (AD++), B27 (all Thermo Fisher), 50% Wnt, 10% R-spondin, 10% noggin conditioned media, 1.25 mM N-acetyl-cysteine (Sigma-Aldrich), 100 ng/mL Primocin (Invivogen), 1 nM (gastric) or 10 nM (intestinal) Gastrin I (Tocris), 50 ng/mL EGF (Peprotech), 2 µM (gastric) or 0.5 µM TGF-beta inhibitor (Tocris), 100 ng/mL FGF-10 (only gastric, Peprotech), 50 ng/mL IGF-1 (only intestinal, Biolegend), 50 ng/mL FGF-basic (only intestinal, Biolegend).

List of 3D organoid expansion and 2D organoid-derived monolayer differentiation medium composition.

**3D organoid expansion media composition**		
Medium components	Gastric media	Colon/Jejunum media
AD DMEM /F12 (12634028, Thermo Fisher Scientific)	30% of total volume	30% of total volume
1X GlutaMAX-I (35050-038, Thermo Fisher Scientific)		
10mmol/L HEPES (15630056, Thermo Fisher Scientific)		
WNT conditioned media	50% of total volume	50% of total volume
R-spondin conditioned media	10% of total volume	10% of total volume
Noggin conditioned media	10% of total volume	10% of total volume
B27 (12587010, Thermo Fischer Scientific)	1X	1X
N-acetyl-cysteine (A9165-25G, Sigma-Aldrich)	1.25mM	1.25mM
Primocin (Ant-pm-1, Invivogen)	100 ng/mL	100 ng/mL
Gastrin-I (3006, Tocris)	1 nM	10 nM
EGF (AF-100-15, Peprotech)	50 ng/mL	50 ng/mL
FGF-10 (100-26, Peprotech)	100 ng/mL	-
A83-01 (A-83-01, Tocris)	2 µM	0.5 µm
IGF-1 (590904, Biolegend)	-	100 ng/mL
FGF-basic (C100-18B, Peprotech)	-	50 ng/mL
**2D organoid-derived monolayer differentiation media composition**		
Medium components	Gastric DIF media	Colon/ Jejunum DIF media
AD DMEM /F12 (12634028, Thermo Fisher Scientific)	80% of total volume	90% of total volume
1X GlutaMAX-I (35050-038, Thermo Fisher Scientific)		
10mmol/L HEPES (15630056, Thermo Fisher Scientific)		

WNT conditioned media	-	-
R-spondin conditioned media	10% of total volume	5% of total volume
Noggin conditioned media	10% of total volume	5% of total volume
B27 (12587010, Thermo Fischer Scientific)	1X	1X
N-acetyl-cysteine (A9165-25G, Sigma-Aldrich)	1.25mM	1.25mM
Primocin (Ant-pm-1, Invivogen)	100 ng/mL	100 ng/mL
Gastrin-I (3006, Tocris)	1 nM	10 nM
EGF (AF-100-15, Peprotech)	50 ng/mL	50 ng/mL
FGF-10 (100-26, Peprotech)	100 ng/mL	-
A83-01 (A-83-01, Tocris)	2 µM	0.5 µm

TCID50 assay

TCID50 assay of supernatant of infected gastrointestinal monolayers was carried out using Vero TMPRSS2 cells. Cells were cultured as described above. The supernatant of infected cells was serially diluted before the inoculation of Vero TMPRSS2 cells. Cells were inoculated with 80 µl of diluted inoculum and incubated for 24h at 37°C and 5% CO2. Cells were fixed with 4% PFA and proceeded to SARS-CoV-2 N protein staining. SARS-CoV-2 positive cells were images and used to determine TCID50.

RT-qPCR for gastrointestinal tissue and organoid-derived monolayers

Total RNA was isolated from gastrointestinal tissue and monolayers using RNeasy Mini Kit (74106, Qiagen) according to the manufacturer's instructions. cDNA was generated using random primers (48190011, Life Technologies) and M-MuLV reverse transcriptase (M0253, New England Biolabs) according to the manufacturer's instructions for monolayers. cDNA for tissue isolated RNA was generated using TaqMan™ Reverse Transcription kit (N8080234, Applied Biosystems). The quantification of RNA levels for tissue origin cDNA was done using SensiFAST™ SYBR® No-ROX Kit (98020, Bioline). Quantitative reverse transcription PCR analysis for monolayer samples was performed using SYBR Green Supermix (172-5270, BioRad) following the manufacturer's instructions, in a CFX96 Touch Real-Time PCR detection system using Bio-Rad CFX Manager software (BioRad) Primers used in this study are listed below. The expression of SARS-CoV-2 entry factors and gastrointestinal cell markers was analyzed using the ΔΔCt method.

List of RT-qPCR assay primers.

Gene	Sequence
GAPDH	(F) 5-CTCTCTGCTCCTCCTGTTCGAC-3
	(R) 5-TGAGCGATGTGGCTCGGCT-3
ACE2	(F) 5-TCAGAACCCTGGACCCTAGC-3
	(R) 5- GTTCTGGTCTTTCAGCCAGGT-3
IFNL2/3	(F) 5-GCCACATAGCCCAGTTCAAG-3
	(R) 5-TGGGAGAGGATATGGTGCAG-3
SARS-CoV-2 N	(F) 5- GCCTCTTCTGTTCCTCATCAC-3
	(R) 5- AGACAGCATCACCGCCATTG-3
18S	(F) 5-TGTGCCGCTAGAGGTGAAATT-3
	(R) 5-TGGCAAATGCTTTCGCTTT-3
SARS-CoV-2 RdRp	(F) 5- GTGARATGGTCATGTGTGGCGG-3
	(R) 5-CARATGTTAAASACACTATTAGCATA-3
IFNL1	(F) 5- GCAGGTTCAAATCTCTGTCAC -3
	(R) 5- AAGACAGGAGAGCTGCAACTC -3
TMPRSS2	(F) 5- CTTTGAACTCAGGGTCACCA -3
	(R) 5- TAGTACTGAGCCGGATGCAC -3
NRP1	(F) 5-GCCACAGTGGAACAGGTGAT -3
	(R) 5-ATGACCGTGGGCTTTTCTGT-3
TMEM106B	(F) 5- TCCACGACCCTGTCCTCG -3

	(R) 5- AGACTTTCCCATGTCGGCAC -3
MUC5AC	(F) 5- CTTCTCAACGTTTGACGGGAAGC-3
	(R) 5- CTTGATCACCACCACCGTCTG -3
MUC2	(F) 5- CGTCCGTCTCCAACATCACC -3
	(R) 5- CACCCTGGTCTCATTGCGAG -3
SI	(F) 5- AATCCTTTTGGCATCCAGAT -3
	(R) 5- GCAGCCAAGAATCCCAAAT -3

Immunofluorescence staining

Gastrointestinal monolayers were seeded on µ-Slide 8 Wells (80826, Ibidi) and cultured under differentiation conditions for 4 days. After differentiation, cells were fixed with 4 % paraformaldehyde for 20 min at RT, permeabilized with 0.5 % Triton X-100 in PBS for 15 min, and then blocked with 1% BSA for 1 h. Cells were stained with ACE2 (1:100, AF933, R&D Systems) MUC5AC (1:100, MA5-12178, Thermo Fischer Scientific), and MUC2 (1:400, sc-15334, Santa Cruz Biotechnology) diluted in blocking buffer overnight at 4°C. Gastrointestinal monolayers were incubated with corresponding secondary antibodies donkey anti-goat Alexa Fluor-488 (1:500, A-11055, Thermo Fisher Scientific), donkey anti-rabbit Alexa Fluor-594(1:500, A-21207, Invitrogen), goat anti-mouse Alexa Fluor-488 (1:500, A-11001, Thermo Fisher Scientific) in blocking solution and incubated at RT for 2 h. Nuclear DNA was stained with Hoechst 33342 (1:5,000; H3570, Life Technologies). Gastrointestinal monolayers were imaged using a Leica SP5 laser scanning confocal microscope (Leica Microsystems) and images were analyzed with Fiji open-source software.

Immunohistochemistry and *In situ* hybridization

Gastrointestinal tissue biopsy samples were fixed using formalin 10% (09122, Neogen) and embedded in paraffin blocks. Embedded tissue sections (5 µm) were routinely processed for histology and immunofluorescence staining as described before[8, 9,10]. Tris-EDTA (10 mM Tris base, 1 mM EDTA solution, pH 9.0) buffer was used for antigen retrieval. Gastrointestinal tissue biopsy samples were probed with MUC2 (1:400, sc-15334, Santa Cruz Biotechnology) and ACE2 (1:100, AF933, R&D Systems) at 4°C overnight, followed by incubation with corresponding secondary antibodies (1:2000, Alexa Fluor488 and Alexa Fluor647, Invitrogen). Nuclear DNA was counter-stained using DAPI (Sigma Aldrich). To examine ACE2 RNA expression in gastrointestinal tissue, fluorescence RNA ISH was carried out using RNAscope Multiplex Fluorescent Reagent Kit v2 (Advanced Cell Diagnostics, Bio-Techne) using available ACE2 probe (probe Hs-ACE2-C2; Advanced Cell Diagnostics), according to the manufacturer's protocol. Immunofluorescence images of stained gastrointestinal tissue were acquired with LSM 780 confocal microscope [(objectives: Plan Apochromat 40x/1.40 oil DIC M27 and Plan Apochromat 10x/0.3 DIC M27), Carl-Zeiss, Jena, Germany]. To reveal tissue and cell morphology, images were combined with Differential Interference Contrast (DIC). All image sets were acquired using equal configurations. Images were processed using ZEN 2012.

Western blot

Organoids were lysed organoids were lysed in RIPA buffer (89900, Thermo Scientific) with protease inhibitor cocktail (11697498001, Roche) (1:50) on ice, followed by sonication. Proteins were separated on SDS–PAGE gels and transferred to the nitrocellulose membrane. Membrane were probed with following antibodies: anti-ACE2 (1:100, AF933, R&D Systems), anti-Tubulin (1:3,000, A2228, Sigma-Aldrich), anti-mouse HRP (1:5000, ab97040, Abcam), anti-goat HRP (1: 5000, sc-2354, Santa Cruz Biotechnology). Protein bands were visualized using luminol (0,25mg/mL, A8511-5G, Sigma-Aldrich) and p-coumaric acid (1.1 mg/mL, sc-215648A, Santa Cruz Biotechnologies) solution. The images were taken using an ImageQuant LAS 4000 CCD camera (GE Healthcare).

RNA-seq analysis

The previously published 3D organoids RNA sequencing data (GEO access number GSE127938) was used to examine the expression of known host factors for SARS-CoV-2 infection using R (4.1.2) and Bioconductor packages[1]. The list of known SARS-CoV-2 infection host factors was compiled using top hits from previously published studies and after filtration 749 genes were used for further

analysis[11-15] (see Table S1). The raw counts of organoid data were normalized using standard DESeq2 analysis and visualized as previously described[1]. The differential expression analysis between corpus, jejunum, and colon organoids was carried out using Benjamini-Hochberg adjusted p-value below 0.05 as the cut-off value. The differential expression data was visualized using the EnhancedVolcano package.

Single cell-RNA sequencing (sc-RNA-seq) data analysis

Publicly available sc-RNA sequencing data were analyzed for the expression of *ACE2, BEST4, CHGA, MUC2, ALPI,* and *ANEP*. Human *ex vivo* small intestine and colon tissue data was analyzed using a gut cell atlas (gutcellatlas.org) containing sc-RNA-seq data of adult intestinal epithelium[6]. Data was visualized using the following settings: age group: adult, category: epithelial, region: largeInt or smallInt. Also, human *ex vivo* ileum sc-RNA-seq data[4] were examined for cell type-specific expression of ACE2 using the Broad Institute single-cell portal (Singlecell. broadinstitute.org). Furthermore, available human *ex vivo* colonic epithelium sc-RNA-seq data[5] (GEO access number GSE116222). was used to visualize the expression of *ACE2, BEST4, CHGA, MUC2,* and *ALPI* as well as *ANEP* using Seurat R package V4[16].

ACE2 overexpression in corpus organoids

ACE2 overexpression in corpus organoids was induced by using a commercially available lentivirus construct of ACE2 (79944, BPS Bioscience). Organoids (patient ID #71) were collected and washed with AD++, centrifugated, and resuspended in TrypLE Express (Gibco). Organoids were incubated for 10 min at 37°C with occasional resuspension with P1000 pipette. Cells were washed with AD++ and pelleted. Cells were resuspended in AD++ supplemented with polybrene (8µg/mL, TR1003G, Thermo Fisher Scientific), RHOKi (10 µM, Y-27632, Sigma-Aldrich) and nicotinamide (10 mM, N0636, Sigma-Aldrich) and seeded into 48-well plate coated with 80µL mix of Matrigel and AD++ (1:2). Cells were transduced with MOI2 of lentivirus and incubated for 16h at 37°C. Following the incubation wells were washed with AD++ and TrypLE Express to collect most of the cells. The collected cells were washed with AD++ and seeded into 3 wells in Matrigel domes and overlayed with complete gastric medium supplemented with Wnt Surrogate-Fc Fusion Recombinant Protein (PHG0403, Gibco) and RHOKi. Organoids were selected with puromycin (1 µg/mL) 48h post-seeding into Matrigel. The puromycin selection was continued for 7 days with full gastric medium supplemented with a Wnt surrogate. Surviving organoids were expanded and characterized for ACE2 expression in differentiated 2D monolayers using immunofluorescent staining.

Mass spectrometry sample preparation

Gastrointestinal organoids were cultured for 8 days after splitting in gastric and EIF expansion medium before collection for protein extraction. GI organoids were lysed in RIPA buffer (Thermo Scientific, 89900) with protease inhibitor cocktail (11697498001, Roche) on ice. Cell debris was removed (2500 rpm for 5 min) and protein concentration was determined (Pierce, 23225). A volume corresponding to 50 µg protein was transferred and topped up to 60 µl with RIPA buffer. Lysates were processed manually using the SP3 protocol as previously described[17] with one-step reduction and alkylation. Briefly, 10 µl of reduction and alkylation buffer (40 mM TCEP, 160 mM CAA, 200 mM ABC, 4% SDS) were added, and samples were incubated at 56 °C for 30 min and cooled to RT. To bind the proteins, 500 µg of paramagnetic beads (1:1 ratio of hydrophilic/hydrophobic beads (GE Healthcare, PN 45152105050250, 65152105050250) were added and proteins were precipitated by adding ACN to a final concentration of 70%. Samples were washed twice with 80% EtOH and once with 100% ACN before reconstitution in 35 µl 100 mM ABC. Samples were digested overnight at 37 °C with Trypsin/Lys-C mix (Promega, V5072) at a protein: enzyme ratio of 50:1 (w/w). The reaction was stopped by adding formic acid to a final concentration of 0.1%. The resulting tryptic peptides were purified using C18-based (Affinisep, AttractSPE) stage tips as described preveliously[18] and dried before resuspension in 0.1% formic acid. The peptide concentration was determined (Pierce, 23290), insoluble particles were removed by centrifugation, samples were transferred to a new plate, and frozen at -80 °C until analysis by LC-MS.

Liquid chromatography-mass spectrometry

LC–MS analysis was conducted on an EVOSEP One system coupled to a Bruker TimsTOF PRO2 mass spectrometer. Five hundred nanograms of sample material were loaded onto the Evotip according to the manufacturer's protocol. Liquid chromatography was performed using the EVOSEP 15 SPD LC method (88 min gradient) with an EV1137 performance column (15 cm × 150 µm, 1.5 µm) at 40 °C, coupled to a 10 µm Zero Dead Volume Captive Spray Emitter (Bruker #1865691). For acquisition in dia-PASEF mode, the acquisition scheme covered the mass range m/z 400–1,201 and ion mobility range 1/K0 0.6–1.6, using 16 frames, with two precursor isolation windows per frame (m/z 26 window width, m/z 1.0 overlap between adjacent windows). Accumulation and ramp times were set to 100 ms. The mass spectrometry proteomics data have been deposited to the ProteomeXchange Consortium via the PRIDE[19] partner repository with the dataset identifier PXD044789.

Proteomics mass spectrometry data analysis

The raw proteomic mass spectrometry data was processed with *DIA-NN* version *1.8* [20] using default settings. MS1 and MS2 mass accuracies were fixed to 20 ppm and a spectral library was generated from the *H. sapiens* Proteome UP000005640_9606 downloaded from UniProt[21]. All following proteomics analysis steps were performed using Python 3.9.7. Precursors were filtered for q-values < 0.01 (on sample, global, and library levels), and only quantified proteotypic precursors were considered for analysis. Afterward, precursors were present in less than 2/3 of the samples, and proteins quantified by less than 2 precursors were excluded. Following this, precursor intensity distributions were normalized using median polish normalization on precursors present in 90% of all samples, where every precursor was multiplied by a sample-specific factor given by the ratio of the median of the sample medians of precursor intensities and the median of precursor intensities in a given sample. For imputing missing data, we first distinguished missingness not at random from missingness at random by counting the number of precursors originating from every protein. If no precursor for a given protein was identified, the precursor value was set to zero, otherwise, the *k-nearest-neighbors* algorithm from the *scikit-learn* package *version 1.2.2* [22] was applied to log2-scaled precursors using *k=2*. Last, precursors were summarized to proteins using the MaxLFQ algorithm as implemented in QuantUMS[23].
Sample quality was assessed during all data processing steps by comparing the numbers of identified precursors, precursor intensity distributions, normalization factors obtained from median polish normalization, and statistical parameters from the DIA-NN report such as signal and median mass accuracies on MS1 and MS2 as well as the average number of missed tryptic cleavages. Additionally, technical quality was assessed by comparing samples to three sample pools containing aliquots of all biological samples. One sample (Corpus #72) did not meet the quality of the other samples and was therefore excluded. Finally, the data processing pipeline was then executed again only on samples satisfying the quality standards excluding the quality control samples.
Clustermaps with proteomic profile correlations and hierarchical clustering of samples were created with the clustermap function from the Python *seaborn* package version *0.11.2* [24]. For differential analysis between corpus, colon, and jejunum organoids Welch tests from the S*ciPy* package version *1.10.1* on log2 protein MaxLFQ abundances with Benjamini-Hochberg multiple testing correction from the *statsmodels* package version *0.13.5* [25] were applied, and plotted against protein mean fold changes. Differentially expressed genes were identified as proteins with absolute mean log2 fold changes between two organoid groups larger than 1 as well as an adjusted p-value below 0.05.

Differential detection of proteins (Figure 2 G) was performed on data before the exclusion of precursors with excessive missing values and before the exclusion of proteins identified by less than two precursors. A protein was considered detected as long as it was present in at least one organoid sample per group (corpus, colon, or jejunum). The relative abundance of selected proteins (Figure S3B) was calculated as follows – Relative abundance = (MaxLFQ (protein of interest) / maximal detected value protein of interest (MaxLFQ)).

Statistics

Experimental results are presented as mean ± standard error of the mean (SEM) of individual patients. There available organoid lines are listed in figure legends as well as statistical tests used for the analysis. Prism Software (v8, GraphPad) was used to carry out statistical analysis using the threshold of significance at $p < 0.05$.

Supplementary References:

1. Kayisoglu, O. et al. (2021). Location-specific cell identity rather than exposure to GI microbiota defines many innate immune signalling cascades in the gut epithelium. Gut. 70(4):687-697. doi: 10.1136/gutjnl-2019-319919.

2.Cantuti-Castelvetri, L. et al. (2020). Neuropilin-1 facilitates SARS-CoV-2 cell entry and infectivity. Science, 370(6518), pp. 856–860. doi: 10.1126/science.abd2985.

3. Baggen, J. et al. (2023). TMEM106B is a receptor mediating ACE2-independent SARS-CoV-2 cell entry. Cell. Aug 3;186(16):3427-3442.e22. doi: 10.1016/j.cell.2023.06.005.

4. Ziegler, C. G. K. et al. (2020).SARS-CoV-2 Receptor ACE2 Is an Interferon-Stimulated Gene in Human Airway Epithelial Cells and Is Detected in Specific Cell Subsets across Tissues. Cell. 181(5):1016-1035.e19. doi: 10.1016/j.cell.2020.04.035.

5. Parikh, K. et al. (2019). Colonic epithelial cell diversity in health and inflammatory bowel disease. Nature, 567(7746):49-55.doi: 10.1038/s41586-019-0992-y.

6. Elmentaite, R. et al. (2021). Cells of the human intestinal tract mapped across space and time. Nature, 597(7875):250-255. doi: 10.1038/s41586-021-03852-1.

7. Peterhoff et al. (2021). A highly specific and sensitive serological assay detects SARS-CoV-2 antibody levels in COVID-19 patients that correlate with neutralization. Infection. 49(1):75-82. doi: 10.1007/s15010-020-01503-7.

8. Berg, J. et al,. (2017). Tyk2 as a target for immune regulation in human viral/bacterial pneumonia. Eur Respir J 50(1):1601953. doi: 10.1183/13993003.01953-2016.

9. Hocke A.C. et al (2013). Emerging human Middle East respiratory syndrome coronavirus causes widespread infection and alveolar damage in human lungs. Am J Respir Crit Care Med. 188(7):882-6. doi: 10.1164/rccm.201305-0954LE.

10. Hönzke, K. et al. (2022). Human lungs show limited permissiveness for SARS-CoV-2 due to scarce ACE2 levels but virus-induced expansion of inflammatory macrophages. Eur Respir J 60(6):2102725. doi: 10.1183/13993003.02725-2021

11.Wang, R. et al. (2021). Genetic Screens Identify Host Factors for SARS-CoV-2 and Common Cold Coronaviruses. Cell. 184(1):106-119.e14. doi: 10.1016/j.cell.2020.12.004.

12. Schneider, W. M. et al. (2021). Genome-Scale Identification of SARS-CoV-2 and Pan-coronavirus Host Factor Networks. Cell. 184(1):120-132.e14. doi: 10.1016/j.cell.2020.12.006.

13. Daniloski, Z. et al. (2020).Identification of required host factors for SARS-CoV-2 infection in human cells.Cell. 184(1):92-105.e16. doi: 10.1016/j.cell.2020.10.030.

14. Schmidt, N. et al. (2020). The SARS-CoV-2 RNA–protein interactome in infected human cells. Nat Microbiol. 6(3):339-353. doi: 10.1038/s41564-020-00846-z.

15. Wei, J. et al. (2020). Genome-wide CRISPR Screens Reveal Host Factors Critical for SARS-CoV-2 Infection. Cell. 184(1):76-91.e13. doi: 10.1016/j.cell.2020.10.028.

16. Hao, Y. et al. (2021). Integrated analysis of multimodal single-cell data. Cell. 184(13):3573-3587.e29. doi: 10.1016/j.cell.2021.04.048.

17. Müller T, et al., (2020). Automated sample preparation with SP3 for low-input clinical proteomics. J. Mol Syst Biol. 16(1):e9111.doi: 10.15252/msb.20199111.

18. Rappsilber J, Mann M, Ishihama Y. (2017). Protocol for micro-purification, enrichment, pre-fractionation and storage of peptides for proteomics using stagetips. Nature Protocols. 2(8):1896-906. doi: 10.1038/nprot.2007.261.

19. Perez-Riverol Y. et al., (2022). The PRIDE database resources in 2022: A Hub for mass spectrometry-based proteomics evidences. Nucleic Acids Res 50 (D1):D543-D552. doi: 10.1093/nar/gkab1038.

20. Demichev, V. et al., (2020). DIA-NN: neural networks and interference correction enable deep proteome coverage in high throughput. Nature methods, 17(1):41-44. doi: 10.1038/s41592-019-0638-x

21. https://www.uniprot.org/proteomes/UP000005640, Access date: 31.01.2022, 12:00 CET.

22. Pedregosa et al., (2011). Scikit-learn: Machine Learning in Python. JMLR 12, pp. 2825-2830.

23. Kistner, F. et al. (2023). QuantUMS: uncertainty minimisation enables confident quantification in proteomics. bioRxiv, 2023-06.

24. Waskom, M. L. (2021). Seaborn: statistical data visualization. Journal of Open Source Software, 6(60), 3021.

25. Seabold, Skipper, and Josef Perktold. (2010). statsmodels: Econometric and statistical modeling with python. Proceedings of the 9th Python in Science Conference.
