# Supplementary material for: SARS-CoV-2 tropism to intestinal but not gastric epithelial cells is defined by limited ACE2 expression
Source: Stem Cell Reports. 2024 Apr 25;19(5):629–38. doi: 10.1016/j.stemcr.2024.03.008 (PMC11103887; doi:10.1016/j.stemcr.2024.03.008)
Supplement: Document S2. Article plus supplemental information [file mmc4.pdf]

Stem Cell Reports
Report
OPEN ACCESS

SARS-CoV-2 tropism to intestinal but not gastric epithelial cells is defined by limited ACE2 expression

Mindaugas Paužuolis,[1] Diana Fatykhova,[2] Boris Zühlke,[3] Torsten Schwecke,[4] Mastura Neyazi,[1] Pilar Samperio-Ventayol,[5,6] Carmen Aguilar,[1] Nicolas Schlegel,[7] Simon Dökel,[8] Markus Ralser,[3,9,10] Andreas Hocke,[3] Christine Krempl,[11] and Sina Bartfeld[1,5,6,*]

[1]Research Centre for Infectious Diseases, Institute for Molecular Infection Biology, Julius Maximilians Universität Würzburg, Würzburg, Germany
[2]Department of Infectious Diseases, Respiratory Medicine and Critical Care, Charité - Universitätsmedizin Berlin, Corporate Member of Freie Universität Berlin and Humboldt-Universität zu Berlin, Berlin, Germany
[3]Institute of Biochemistry, Charité - Universitätsmedizin Berlin, Berlin, Germany
[4]Core Facility for High-Throughput Mass Spectrometry, Institute of Biochemistry, Charité - Universitätsmedizin Berlin, Berlin, Germany
[5]Si-M/'Der Simulierte Mensch', Technische Universität Berlin and Charité–Universitätsmedizin Berlin, Berlin, Germany
[6]Department of Medical Biotechnology, Institute of Biotechnology, Technische Universität Berlin, Berlin, Germany
[7]Department of General, Visceral, Transplant, Vascular and Pediatric Surgery, University Hospital Würzburg, Würzburg, Germany
[8]Institute of Veterinary Pathology, Freie Universität Berlin, Berlin, Germany
[9]The Francis Crick Institute, Molecular Biology of Metabolism Laboratory, London, UK
[10]The Wellcome Centre for Human Genetics, Nuffield Department of Medicine, University of Oxford, Oxford, UK
[11]Institute for Virology and Immunobiology, Julius Maximilian University of Würzburg, Würzburg, Germany
*Correspondence: s.bartfeld@tu-berlin.de
https://doi.org/10.1016/j.stemcr.2024.03.008

SUMMARY

Severe acute respiratory syndrome coronavirus 2 (SARS-CoV-2) infection primarily affects the lung but can also cause gastrointestinal (GI) symptoms. *In vitro* experiments confirmed that SARS-CoV-2 robustly infects intestinal epithelium. However, data on infection of adult gastric epithelium are sparse and a side-by-side comparison of the infection in the major segments of the GI tract is lacking. We provide this direct comparison in organoid-derived monolayers and demonstrate that SARS-CoV-2 robustly infects intestinal epithelium, while gastric epithelium is resistant to infection. RNA sequencing and proteome analysis pointed to angiotensin-converting enzyme 2 (ACE2) as a critical factor, and, indeed, ectopic expression of ACE2 increased susceptibility of gastric organoid-derived monolayers to SARS-CoV-2. ACE2 expression pattern in GI biopsies of patients mirrors SARS-CoV-2 infection levels in monolayers. Thus, local ACE2 expression limits SARS-CoV-2 expression in the GI tract to the intestine, suggesting that the intestine, but not the stomach, is likely to be important in viral replication and possibly transmission.

INTRODUCTION

Over the last two decades, several viruses of zoonotic origin from the *Coronaviridae* family have become a global health concern. Severe acute respiratory syndrome-related coronavirus (SARS-CoV) and Middle East respiratory syndrome coronavirus (MERS-CoV) caused outbreaks of severe respiratory diseases, and severe acute respiratory syndrome coronavirus 2 (SARS-CoV-2) caused the global pandemic of COVID-19 (Zhu et al., 2020).

Severity of COVID-19 is highly variable and ranges from asymptomatic infections to severe respiratory system failure and multi-organ failure. Symptoms mainly concern the respiratory tract yet can also include diarrhea and vomiting, pointing to an involvement of the gastrointestinal (GI) tract (Neurath, 2020). On the molecular level, sequencing and histology data showed that the SARS-CoV-2 entry receptor, angiotensin-converting enzyme 2 (ACE2), is highly expressed in the GI tract at RNA and protein levels (Hikmet et al., 2020; Qi et al., 2020).

Organoids, 3-dimensional primary cell cultures derived from stem cells, and organoid-derived monolayers have been used to study SARS-CoV-2 infection and cell-type tropism. In these studies, SARS-CoV-2 robustly infects small intestinal cells and colonic cells, and to a certain extent also pediatric and fetal gastric cells (Zhou et al., 2020; Lamers et al., 2020; Stanifer et al., 2020; Giobbe et al., 2021; Heuberger et al., 2021). Immunofluorescence imaging pointed to SARS-CoV-2 tropism to enterocytes (Lamers et al., 2020; Zhou et al., 2020). In fetal and pediatric gastric organoids, SARS-CoV-2 showed tropism to somatostatin-positive enteroendocrine cells (Giobbe et al., 2021).

While a general involvement of the GI tract in SARS-CoV-2 infection is clear, most of the studies focused on individual segments of GI tract such as stomach, ileum, or colon, and there is a lack of direct side-by-side comparisons of SARS-CoV-2 entry factors and viral infection in the major GI tract segments.

Here, using adult stem cell-derived organoids and their derived monolayers, we examine SARS-CoV-2 infection in the gastric (corpus), small intestinal, and colonic epithelium. While small intestinal epithelium and colonic epithelium were susceptible, the gastric epithelium was

This is an open access article under the CC BY license (http://creativecommons.org/licenses/by/4.0/).

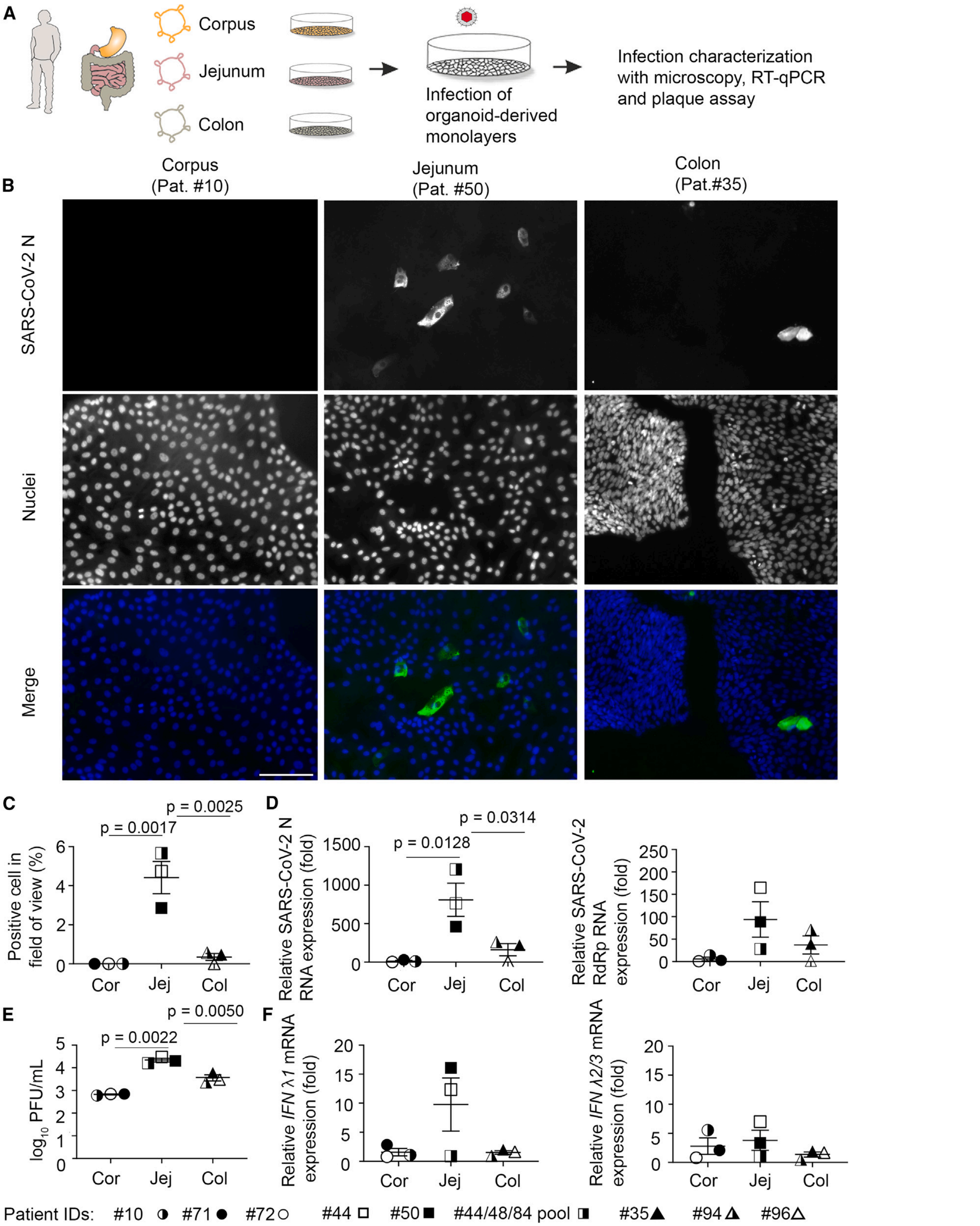

(legend on next page)

protected from infection. This was mirrored in the expression of ACE2. Ectopic expression of ACE2 in corpus monolayers increased the susceptibility to SARS-CoV-2 infection. Our results indicate that SARS-CoV-2 tropism in GI tract is limited by localized expression of ACE2 in the GI tract.

RESULTS

SARS-CoV-2 shows differential infection tropism in GI organoid-derived monolayers

To compare the infection in the three major GI segments stomach, small intestine, and colon, we focused on the large regions corpus, jejunum, and colon. Gastric, jejunal, and colonic organoids were expanded and seeded into monolayers, and monolayers were differentiated for 4 days. Differentiated monolayers expressed regional markers as shown before (Stanifer et al., 2020; Aguilar et al., 2022; Figure S1). Monolayers were infected with 1 x 10^6 plaque-forming units (PFUs) of SARS-CoV-2 and examined 24 h post-infection (hpi) (Figure 1A). Immunofluorescence staining and RT-qPCR for SARS-CoV-2 nucleocapsid (N) protein and RT-qPCR for viral RNA-dependent polymerase (RdRp) indicated that jejunal cells were most susceptible to SARS-CoV-2 infection; colonic cells were also susceptible, but infection was not detected in gastric cells (Figures 1B–1D). Plaque assay, which measures infectious virus in the supernatant of infected cells, showed significantly higher number of viral particles (2.5×10^4 PFU/mL) in jejunal than colonic monolayers (3.75×10^3 PFU/mL) and corpus monolayers (6.333×10^2 PFU/mL) (Figure 1E), indicating most productive infection in the jejunum. Host innate type III interferon response was most pronounced in jejunal monolayers (Figure 1F). Together, this indicated a preferential tropism of SARS-CoV-2 for small intestinal cells.

ACE2 is differentially expressed between GI segments

To understand the mechanism underlying the difference in infection in the GI segments, we used our existing dataset of RNA expression in organoids (Kayisoglu et al., 2021) and focused on the 3 major regions corpus, jejunum, and colon. RNA expression profiles of the 3 GI segments clustered together (Figure 2A). Region-specific markers were expressed as expected (Figure S2). To search for host factors that may influence SARS-CoV-2 infection in the GI tract, we generated a list of host factors identified by CRISPR screens (Table S1). Common genes in the list of host factors and differentially expressed genes (DEGs) between stomach and both intestinal segments, in our dataset, included *ACE2* (Figure 2B; Table S2). To verify these data on protein level, we performed proteome analysis of the organoids from the corpus, jejunum, and colon. Protein expression profiles of organoids clustered together according to the GI segment they were derived from (Figure 2C). Differential expression analysis of the proteome using the same list of host factors as for RNA sequencing analysis highlighted ACE2 among other proteins (Figure 2D), and ACE2 was one of only five proteins that were expressed commonly in the jejunum and colon, but not in the corpus (Figure 2E; Tables S3 and S4). Western blot of ACE2 confirmed the relative abundance of ACE2 in intestinal organoids compared to no detectable protein in corpus organoids (Figure 2F). Immunofluorescence staining of ACE2 also showed that the protein is not detectable in gastric organoid-derived monolayers (Figure 2G). Additional SARS-CoV-2 entry factors transmembrane serine protease 2 (*TMPRSS2*), Neuropilin 1 (*NRP1*), and transmembrane protein 106B (*TMEM106B*) were expressed in the gastric organoids and organoid-derived monolayers and thus are unlikely to be the limiting factor (Figure S3). We concluded that ACE2 is not expressed in gastric corpus organoids or organoid-derived monolayers. We hypothesized that the lack of this main entry receptor for SARS-CoV-2 may underlie the protection of the gastric organoid-derived monolayers from infection with this virus.

Lentiviral ACE2 expression in corpus organoids increases susceptibility to SARS-CoV-2 infection

To analyze, whether ACE2 expression is the limiting factor for SARS-CoV-2 infection in corpus monolayers, we generated corpus organoid lines ectopically expressing ACE2 ($ACE2^+$ corpus) (Figure 3A). Expression of ACE2 in the

Figure 1. SARS-CoV-2 infects small intestinal and colonic, but not gastric, organoid-derived monolayers
(A) Scheme of experimental setup. Organoid-derived monolayers were differentiated for 4 days before incubation with 1×10^6 PFU of SARS-CoV-2. The infection was characterized 24 hpi.
(B) SARS-CoV-2 nucleocapsid (N) protein (green) stained by immunofluorescence. Nuclei were stained with Hoechst 33342. Scale bar, 100 μm.
(C) Quantification of the staining shown in (B). Ten images were quantified per donor.
(D) SARS-CoV-2 N and RdRp RNA quantification using RT-qPCR in monolayer lysates. Data were normalized to *18S* ribosomal RNA.
(E) Quantification of infectious virus particles in the supernatant of infected monolayers using plaque assay.
(F) Type III interferon (IFNλ) *IFNλ1* and *IFNλ2/3* RNA quantification in infected monolayer lysates.
Data in (C)–(F) presented as mean of 3 individual donors or pools as indicated ±SEM. Statistical analysis was carried out using one-way ANOVA with Tukey's multiple comparisons test.

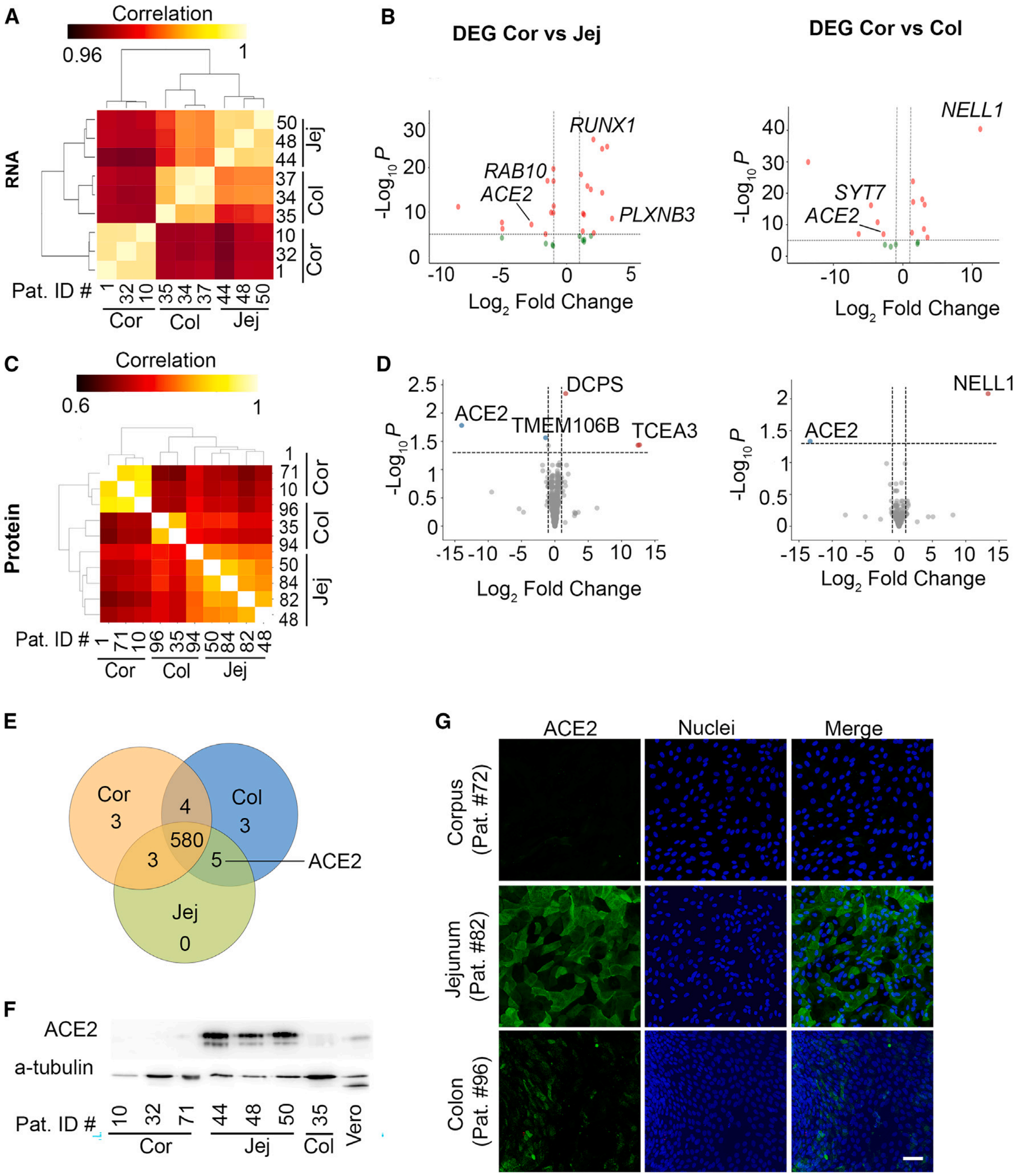

Figure 2. ACE2 is not expressed in corpus organoids and organoid-derived monolayers

(A) Published data were re-analyzed (Kayisoglu et al., 2021) to show hierarchical clustering of total RNA transcriptome of corpus, jejunum, and colon organoids.

(B) Volcano plot visualizing genes associated with SARS-CoV-2 infection by published CRISPR screens and differentially expressed in corpus and jejunum (left) or corpus and colon (right) organoids. Red dots visualize DEG with $p \leq 0.05$ and ≥ 2 log2 fold change. Green dots visualize DEG with $p \geq 0.05$ and ≤ 2 log2 fold change. See Table S2.

(C) Hierarchical clustering of corpus, jejunum, and colon 3D organoids total proteome.

(D) Volcano plot visualizing proteins associated with SARS-CoV-2 infection by CRISPR screens and differentially expressed in corpus and jejunum (left) or corpus and colon (right) organoids. Blue dots visualize proteins downregulated with $p \leq 0.05$ and $\leq$-1 log2 fold change. Red dots identify upregulated proteins with $p \leq 0.05$ and ≥ 1 log2 fold change. See Table S3.

(legend continued on next page)

transduced cells was well observable by immunofluorescence staining, albeit still lower than ACE2 expression in jejunal cells (Figure 3B). Three ACE2-expressing lines of corpus organoids of one patient (#71) were established and infected with SARS-CoV-2 as earlier. Immunofluorescence microscopy for SARS-CoV-2 nucleocapsid protein (N) indicated that $ACE2^+$ corpus organoid-derived monolayers were susceptible to SARS-CoV-2 infections (Figure 3C) with infection rate of 9.6%–11.6% (Figure 3D). Quantification of SARS-CoV-2 nucleocapsid (N) RNA by RT-qPCR confirmed this susceptibility (Figure 3E). Plaque assay showed significantly higher number of infectious virus particles in the supernatant of $ACE2^+$ corpus cells, indicating productive virus replication (Figure 3F), yet the assay also showed some infectious virus in the supernatant of the wild-type (WT) corpus cells, as was already observed in Figure 1E. We suspected that these were residual virus particles from the initial high infection dose that generally escape washing steps. To address this point, we examined SARS-CoV-2 growth in $ACE2^+$ corpus organoid-derived monolayers over time. The viral N RNA started to increase 8 hpi and peaked at 24 hpi. The later time point of 48 hpi showed an increase in SARS-CoV-2 RdRp RNA levels (Figures 3G and 3H). Using tissue culture infection dose $(TCID)_{50}$ assay we observed increased virus release at 24 hpi, which remained stable until 48 hpi (Figure 3I).

In conclusion, ectopic ACE2 expression in corpus organoid-derived monolayers resulted in increased susceptibility to SARS-CoV-2 infection, indicating that the absence of ACE2 is the central factor preventing infection of human gastric epithelium.

ACE2 expression in GI tissue mirrors expression in organoids

To verify the expression pattern of ACE2 in human tissue, we used RNAscope and immunofluorescence staining in GI tissue biopsies and RT-qPCR of *ex vivo*-isolated epithelium crypts. ACE2 was not detectable at protein or RNA level in gastric glands. The highest ACE2 expression and abundance of *ACE2* mRNA were observed in jejunal sections, particularly the villus regions (Figure 4A). In the colon, ACE2 expression was restricted to a few cells and at lower intensities as compared to the jejunum. In some cells, the *ACE2* RNA signal does not fully correspond to protein expression. MUC2, which marks intestinal goblet cells, was used as a control staining, and, while negative in the corpus, as expected, it also showed that ACE2 was absent from MUC2-positive goblet cells. In the colon, where goblet cells are highly abundant, only few MUC2-negative cells expressed apical ACE2 protein (Figure 4A, arrowhead). Analysis of published datasets from single-cell RNA sequencing of human small and large intestine shows that the *ACE2*-expressing population overlaps with populations expressing markers of enterocytes (Figure S4). RT-qPCR of tissue-isolated total RNA showed again the highest *ACE2* mRNA levels in jejunum tissue, compared to mRNA levels in colon or corpus tissue, but it did not pick up a difference between the corpus and colon (Figure 4B). As control, we also examined *TMPRSS2* expression, which was expressed throughout the GI tract as measured by RT-qPCR (Figure 4B). These matched the expression patterns identified in transcriptomic and proteomic data analysis (Figure S3). To allow a direct comparison of organoids, their derived monolayers, and differentiated monolayers and tissue, we probed *ACE2* using RT-qPCR in jejunum samples. We observed that differentiation of monolayers strongly increased *ACE2* expression (Figure 4C), matching to the observation that crypts did not show *ACE2* mRNA in the in situ hybridisation (ISH).

Together, the data indicated that the expression of ACE2 that we observed in organoids and organoid-derived monolayers was mirroring the expression in the tissue: the corpus was devoid of ACE2, the jejunum expresses high levels of ACE2, especially the non-secretory cells of the villus, and the colon expresses lower levels of ACE2. We conclude that the infection of SARS-CoV-2 is restricted by the presence of ACE2 on GI epithelial cells.

DISCUSSION

Clinical reports have pointed to a GI component in COVID-19 disease: approximately 10%–30% of patients present with diarrhea (Neurath, 2020), and the virus is detected in stool samples (Zhang et al., 2020; Zhou et al., 2020). In the present study, we performed a side-by-side comparison of organoid-derived monolayers from the three major segments of the GI tract. We find that the virus infection is strongest in jejunal and not detectable in gastric epithelial cells. We identify the virus entry receptor ACE2 as the limiting factor for infection in gastric cells.

The importance of GI infection in COVID-19 is not yet understood. A large multicenter study reported that GI symptoms correlate with lower severity of COVID-19 (Livanos et al., 2021), but specific populations may also be correlated with higher severity of COVID-19 (Jin et al., 2020).

(E) Venn diagram of host proteins associated with SARS-CoV-2 infection. See Table S4.

(F) ACE2 expression analysis using western blot in organoids and Vero cell line as control.

(G) Immunostaining of ACE2 (green) in differentiated organoid-derived monolayers. Nuclei stained with Hoechst 33342. Scale bar is 30 μm.

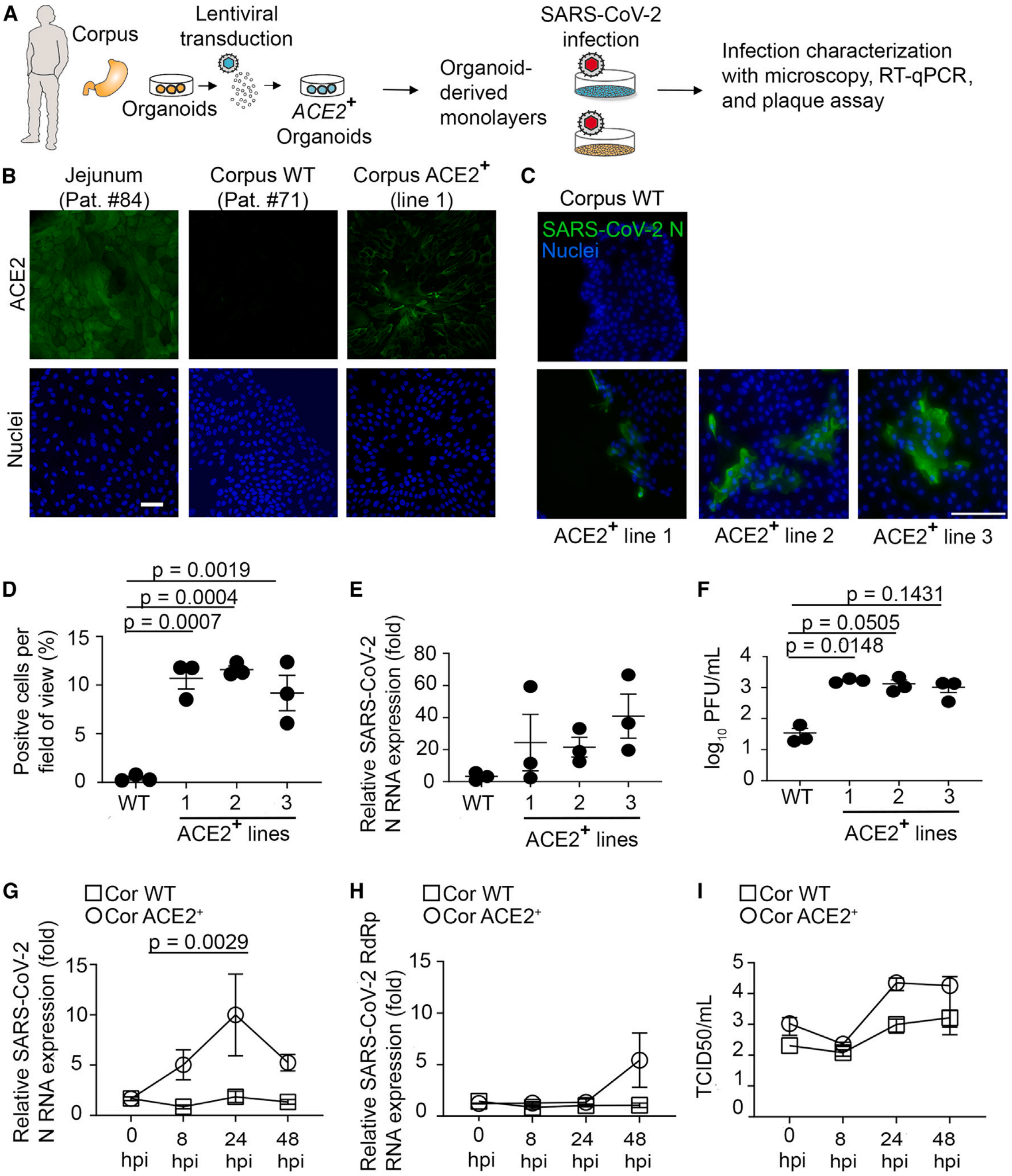

Figure 3. Ectopic ACE2 expression increases the susceptibility of differentiated corpus monolayers for SARS-CoV-2 infection

(A) Scheme of experimental setup. Parental line (wild-type [WT]) and ectopically ACE2-expressing corpus ($ACE2^+$ corpus) lines are compared. Organoid-derived monolayers were differentiated for 4 days before infection with 1×10^6 PFU of SARS-CoV-2. Infection was characterized at 24 hpi.

(B) Immunostaining of ACE2 expression. Scale bar is 30 μm.

(C) Immunostaining of SARS-CoV-2 N protein. Scale bar is 100 μm.

(D) Quantification of immunofluorescence shown in C. Ten images were quantified per line.

(E) Quantification of SARS-CoV-2 N RNA by RT-qPCR normalized to *GAPDH*.

(F) Quantification of infectious virus particles in the supernatant by plaque assay.

(G) SARS-CoV-2 N RNA quantification at different time points after infection using RT-qPCR. Data were normalized to *GAPDH*.

(legend continued on next page)

Thus, depending on the study, it was speculated that GI symptoms may attenuate inflammatory response to SARS-CoV-2, thus leading to a milder disease (Livanos et al., 2021), or that GI symptoms may lead to higher electrolyte disturbances, aggravating disease (Neurath, 2020). Therefore, the importance of GI infection in the course of the disease remains unclear and deserves further investigation.

The data presented here corroborate findings that SARS-CoV-2 can robustly infect small intestinal and colon epithelium. Similarly to published studies, we observed production of viral RNA and viral protein in infected cells (Lamers et al., 2020; Zang et al., 2020; Beumer et al., 2021; Zhao et al., 2021). In our data, compared to jejunum, colon organoid-derived monolayers were less susceptible to infection, which correlated with lower ACE2 expression levels. The staining in tissue sections demonstrate that, in the colon, ACE2 is only expressed by a subpopulation of cells, which are not goblet cells. Available single-cell transcriptomic data indicate that absorptive enterocytes are expressing the highest levels of *ACE2* (Figure S4). One other report has compared 12 lines of terminal ileum and 13 lines of ascending colon and found very high variation of ACE2 expression between the individual lines, but no statistically significant difference between the ileum and colon (Jang et al., 2022). The use of different expansion media, either promoting expansion of undifferentiated cells (Jang et al., 2022) or promoting also expansion of secretory cells (Fujii et al., 2018), which we use here, influences the presence of goblet cells and thus likely also susceptibility to SARS-CoV-2 infection. The comparison with patient tissue suggests that indeed ACE2-expressing cells are much less prominent in the colon than in the jejunum.

The data presented here show no expression of ACE2 in the stomach and in gastric organoids. During the early stages of the SARS-CoV-2 pandemic, single case reports showed SARS-CoV-2 infection in gastric tissue as well as in lower regions of the GI tract (Xiao et al., 2020). However, single-cell RNA sequencing data and patient biopsy data later suggested that ACE2 expression in the esophagus and stomach is related to the development of intestinal metaplasia in gastric tissue (Xu et al., 2020; Jin et al., 2021), indicating that healthy gastric epithelium does not express ACE2. The ectopic expression of ACE2 in the corpus, which we perform here, could mimic the situation in patients' pathological conditions such as intestinal metaplasia in gastric or esophagus epithelium. These regions of high ACE2 expression allow SARS-CoV-2 infection as suggested by others (Xu et al., 2020; Jin et al., 2021).

A previous study has focused on fetal and pediatric gastric epithelium and demonstrated some susceptibility to SARS-CoV-2 infection and ACE2 expression in organoids derived from these tissues (Giobbe et al., 2021). In comparison to the fetal and pediatric organoids, adult organoids were poorly infectible, and the classical plaque assay could not detect virus release from the infected cells. It is possible that ACE2 expression changes with age in the GI tract epithelium, as is suggested by higher abundance of ACE2 reads in pediatric or fetal organoids compared to adult organoids in the RNA sequencing data (Giobbe et al., 2021).

Our data underline that SARS-CoV-2 can infect cells within the intestine, but not the stomach. It is likely that the small intestine is the prime site of viral replication.

EXPERIMENTAL PROCEDURES

Resource availability

Lead contact

Further information and requests for resources and reagents should be directed to and will be fulfilled by the corresponding author, Sina Bartfeld (s.bartfeld@tu-berlin.de).

Materials availability

There are restrictions to the availability of human organoid cultures due to the limitations of the consent that was given by the patients and the guidance of the ethical committee.

Data and code availability

The mass spectroscopy proteomics data were deposited to PRIDE with the data identifier PXD044789.

Organoid culture and 2D monolayer seeding

GI tissue biopsies were obtained from donors that underwent surgical resection at the University Hospital of Würzburg (see supplemental experimental procedures). The acquisition of patient material was approved by the ethical committee of the University of Würzburg (Approval 37/16), and informed consent was obtained from all donors. Organoids were generated following previously published protocols (Fujii et al., 2018; Kayisoglu et al., 2021) (see supplemental experimental procedures).

Monolayers were generated from organoids 7 days after seeding. Organoids were collected in Advanced DMEM (AD++), centrifuged at 450 g for 5 min, resuspended in TrypLE Express (Gibco), and mechanically disrupted followed by incubation at 37°C for 10 min. Single-cell suspension was washed and centrifuged at 450 g for 5 min. Single cells were seeded in 48-well plate (833923, Sarstedt) using the expansion media supplemented with Rho kinase

(H) SARS-CoV-2 RdRp RNA quantification at different time points after infection. Data were normalized to *GAPDH*.
(I) Quantification of infectious virus production over time by $TCID_{50}$ assay.
Data in (D), (E), and (F) presented as mean ± SEM of 3 independent experiments. Data in (G), (H), and (I) presented as mean ± SEM of 3 organoid lines. Statistical analysis was carried out using one-way ANOVA with Tukey's multiple comparisons test.

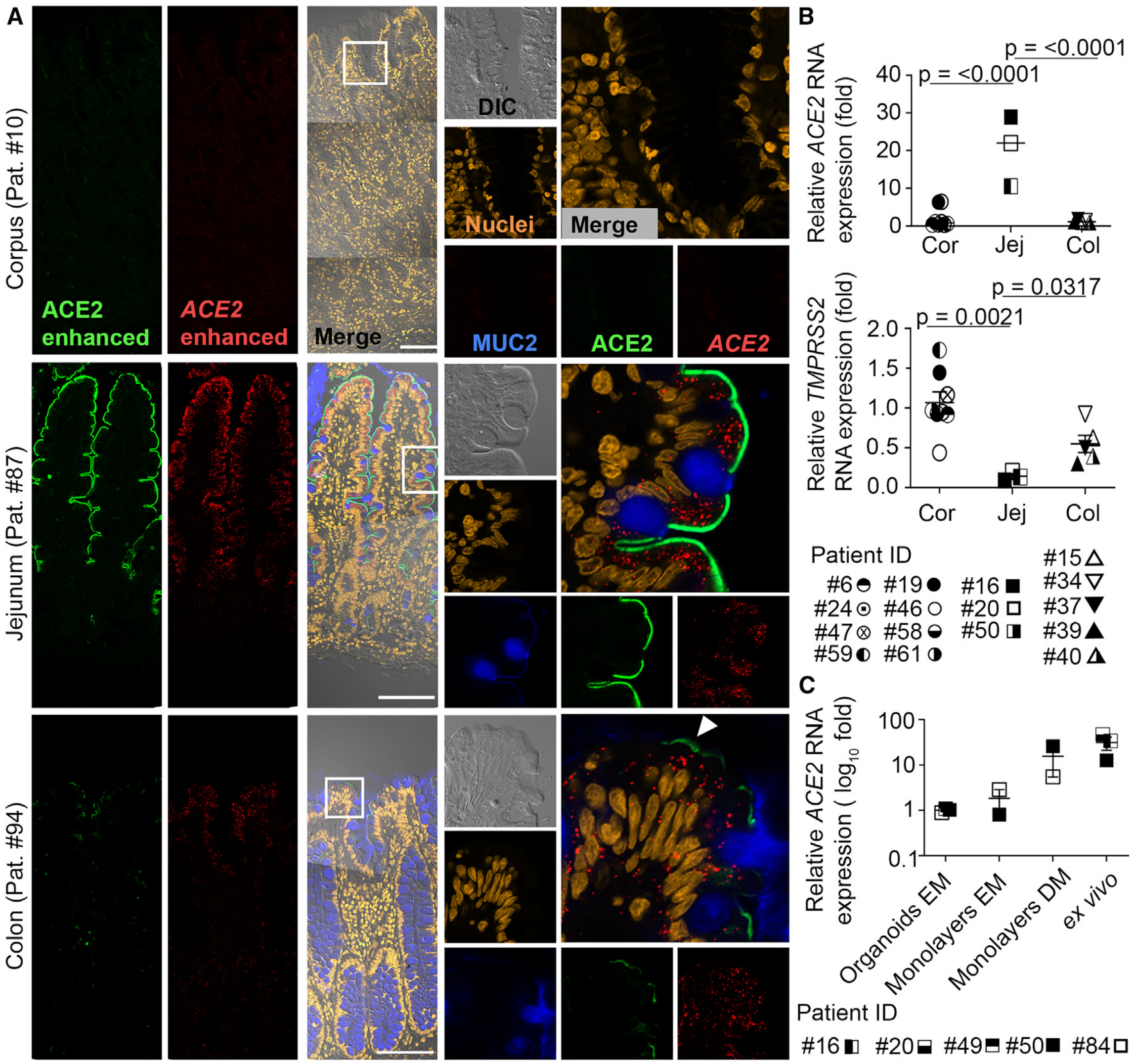

Figure 4. Differential expression of ACE2 in gastrointestinal tissue

(A) Immunostaining of MUC2 protein (blue), ACE2 protein (green), and *in situ* hybridization of *ACE2* mRNA (red) in tissue biopsies. Left column with enhanced green and red intensity to visualize expression in colon. Nuclei were stained with DAPI (yellow). Scale bar is 30 μm. Images are representative of tissues from 2 patients per GI segment.

(B) Relative mRNA levels of *ACE2* (upper panel) and *TMPRSS2* (lower panel) in total RNA from tissue biopsies using RT-qPCR. Data were normalized to *GAPDH* and compared to corpus mRNA levels. Data presented as mean ± SEM of indicated individual donors per GI segment. Statistical analysis was carried out using one-way ANOVA with Tukey's multiple comparisons test.

(C) Relative mRNA levels of *ACE2* in RNA isolations from organoids, organoid-derived monolayers in expansion medium (EM), differentiated monolayers in differentiation medium (DM), and *ex vivo*-isolated epithelium using RT-qPCR. Data were normalized to *GAPDH* and compared to organoid mRNA levels. Data presented as mean ± SEM of individual donors.

inhibitor (RHOKi, 10 μM, Y-27632, Sigma-Aldrich). The following day, expansion media was replaced with differentiation media without Wnt for all GI segments. For intestinal segments, FGF-2, IGF-1, 50% R-Spondin, and 50% Noggin were removed (Stanifer et al., 2020; Aguilar et al., 2022) (see supplemental experimental procedures). Monolayers were grown for 4 days under differentiation condition before the SARS-CoV-2 infection.

Ectopic expression of ACE2 was performed as published before (Hönzke et al., 2022).

SUPPLEMENTAL INFORMATION

Supplemental information can be found online at https://doi.org/10.1016/j.stemcr.2024.03.008.

ACKNOWLEDGMENTS

This work was supported by the University of Wuerzburg (ZINF Young Investigator to S.B.), the DFG (DFG GRK 2157: 3D Tissue

Models for Studying Microbial Infections by Human Pathogens, Project 10, to S.B., DFG SFB-TR84, B6, Z1a to A.H.), BMBF (NUM-COVID 19, Organo-Strat 01KX2021) to S.B., M.R., and A.H., and the Einstein Center 3R to A.H. We thank Stefan Pöhlmann for sharing the Vero E6-TMPRSS2 cells and Steeve Boulant and Megan Stanifer for sharing protocols and methods. We thank Katharina Hellwig for excellent technical assistance.

AUTHOR CONTRIBUTIONS

S.B., M.P., and A.H. conceptualized the project. M.P., D.F., T.S., and S.D. carried out experiments. M.N. contributed to methodology. D.F. carried out tissue imaging. M.P. and B.Z. analyzed datasets. C.A., A.H., C.K., M.R., and S.B. supervised experiments. M.P. and S.B. wrote the original manuscript. All authors reviewed and edited the manuscript.

DECLARATION OF INTERESTS

The authors declare no competing interests.

Received: September 7, 2023
Revised: March 18, 2024
Accepted: March 19, 2024
Published: April 25, 2024

REFERENCES

Aguilar, C., Pauzuolis, M., Pompaiah, M., Vafadarnejad, E., Arampatzi, P., Fischer, M., Narres, D., Neyazi, M., Kayisoglu, Ö., Sell, T., et al. (2022). Helicobacter pylori shows tropism to gastric differentiated pit cells dependent on urea chemotaxis. Nat. Commun. *13*, 1–14. https://doi.org/10.1038/s41467-022-33165-4.

Beumer, J., Geurts, M.H., Lamers, M.M., Puschhof, J., Zhang, J., van der Vaart, J., Mykytyn, A.Z., Breugem, T.I., Riesebosch, S., Schipper, D., et al. (2021). A CRISPR/Cas9 genetically engineered organoid biobank reveals essential host factors for coronaviruses. Nat. Commun. *12*, 1–12. https://doi.org/10.1038/s41467-021-25729-7.

Fujii, M., Matano, M., Toshimitsu, K., Takano, A., Mikami, Y., Nishikori, S., Sugimoto, S., and Sato, T. (2018). Human Intestinal Organoids Maintain Self-Renewal Capacity and Cellular Diversity in Niche-Inspired Culture Condition. Cell Stem Cell *23*, 787–793.e6. https://doi.org/10.1016/J.STEM.2018.11.016.

Giobbe, G.G., Bonfante, F., Jones, B.C., Gagliano, O., Luni, C., Zambaiti, E., Perin, S., Laterza, C., Busslinger, G., Stuart, H., et al. (2021). SARS-CoV-2 infection and replication in human gastric organoids. Nat. Commun. *12*, 1–14. https://doi.org/10.1038/s41467-021-26762-2.

Heuberger, J., Trimpert, J., Vladimirova, D., Goosmann, C., Lin, M., Schmuck, R., Mollenkopf, H.J., Brinkmann, V., Tacke, F., Osterrieder, N., and Sigal, M. (2021). Epithelial response to IFN-γ promotes SARS-CoV-2 infection. EMBO Mol. Med. *13*, e13191. https://doi.org/10.15252/emmm.202013191.

Hikmet, F., Méar, L., Edvinsson, Å., Micke, P., Uhlén, M., and Lindskog, C. (2020). The protein expression profile of ACE2 in human tissues. Mol. Syst. Biol. *16*, e9610. https://doi.org/10.15252/MSB.20209610.

Hönzke, K., Obermayer, B., Mache, C., Fatykhova, D., Kessler, M., Dökel, S., Wyler, E., Baumgardt, M., Löwa, A., Hoffmann, K., et al. (2022). Human lungs show limited permissiveness for SARS-CoV-2 due to scarce ACE2 levels but virus-induced expansion of inflammatory macrophages. Eur. Respir. J. *60*, 2102725. https://doi.org/10.1183/13993003.02725-2021.

Jang, K.K., Kaczmarek, M.E., Dallari, S., Chen, Y.H., Tada, T., Axelrad, J., Landau, N.R., Stapleford, K.A., and Cadwell, K. (2022). Variable susceptibility of intestinal organoid derived monolayers to SARS-CoV-2 infection. PLoS Biol. *20*, e3001592. https://doi.org/10.1371/journal.pbio.3001592.

Jin, R.U., Brown, J.W., Li, Q.K., Bayguinov, P.O., Wang, J.S., and Mills, J.C. (2021). Tropism of SARS-CoV-2 for Barrett's Esophagus may Increase Susceptibility to Developing COVID-19. Gastroenterology *160*, 2165–2168.e4. https://doi.org/10.1053/j.gastro.2021.01.024.

Jin, X., Lian, J.S., Hu, J.H., Gao, J., Zheng, L., Zhang, Y.M., Hao, S.R., Jia, H.Y., Zhang, X.L., Yu, G.D., et al. (2020). Epidemiological, clinical and virological characteristics of 74 cases of coronavirus-infected disease 2019 (COVID-19) with gastrointestinal symptoms. Gut *69*, 1002–1009. https://doi.org/10.1136/gutjnl-2020-320926.

Kayisoglu, O., Weiss, F., Niklas, C., Pierotti, I., Pompaiah, M., Wallaschek, N., Germer, C.T., Wiegering, A., and Bartfeld, S. (2021). Location-specific cell identity rather than exposure to GI microbiota defines many innate immune signalling cascades in the gut epithelium. Gut *70*, 687–697. https://doi.org/10.1136/GUTJNL-2019-319919.

Lamers, M.M., Beumer, J., van der Vaart, J., Knoops, K., Puschhof, J., Breugem, T.I., Ravelli, R.B.G., Paul van Schayck, J., Mykytyn, A.Z., Duimel, H.Q., et al. (2020). SARS-CoV-2 productively infects human gut enterocytes. Science *369*, 50–54. https://doi.org/10.1126/SCIENCE.ABC1669.

Livanos, A.E., Jha, D., Cossarini, F., Gonzalez-Reiche, A.S., Tokuyama, M., Aydillo, T., Parigi, T.L., Ladinsky, M.S., Ramos, I., Dunleavy, K., et al. (2021). Intestinal Host Response to SARS-CoV-2 Infection and COVID-19 Outcomes in Patients With Gastrointestinal Symptoms. Gastroenterology *160*, 2435–2450.e34. https://doi.org/10.1053/J.GASTRO.2021.02.056.

Neurath, M.F. (2020). COVID-19 and immunomodulation in IBD. Gut *69*, 1335–1342. https://doi.org/10.1136/GUTJNL-2020-321269.

Qi, F., Qian, S., Zhang, S., and Zhang, Z. (2020). Single cell RNA sequencing of 13 human tissues identify cell types and receptors of human coronaviruses. Biochem. Biophys. Res. Commun. *526*, 135–140. https://doi.org/10.1016/J.BBRC.2020.03.044.

Stanifer, M.L., Kee, C., Cortese, M., Zumaran, C.M., Triana, S., Mukenhirn, M., Kraeusslich, H.G., Alexandrov, T., Bartenschlager, R., and Boulant, S. (2020). Critical Role of Type III Interferon in Controlling SARS-CoV-2 Infection in Human Intestinal Epithelial Cells. Cell Rep. *32*, 107863. https://doi.org/10.1016/j.celrep.2020.107863.

Xiao, F., Tang, M., Zheng, X., Liu, Y., Li, X., and Shan, H. (2020). Evidence for Gastrointestinal Infection of SARS-CoV-2. Gastroenterology *158*, 1831–1833.e3. https://doi.org/10.1053/j.gastro.2020.02.055.

Xu, J., Chu, M., Zhong, F., Tan, X., Tang, G., Mai, J., Lai, N., Guan, C., Liang, Y., and Liao, G. (2020). Digestive symptoms

of COVID-19 and expression of ACE2 in digestive tract organs. Cell Death Discov. *11*, 76. https://doi.org/10.1038/s41420-020-00307-w.

Zang, R., Gomez Castro, M.F., McCune, B.T., Zeng, Q., Rothlauf, P.W., Sonnek, N.M., Liu, Z., Brulois, K.F., Wang, X., Greenberg, H.B., et al. (2020). TMPRSS2 and TMPRSS4 promote SARS-CoV-2 infection of human small intestinal enterocytes. Sci. Immunol. *5*, eabc3582. https://doi.org/10.1126/sciimmunol.abc3582.

Zhang, Y., Chen, C., Zhu, S., Shu, C., Wang, D., Song, J., Song, Y., Zhen, W., Feng, Z., Wu, G., et al. (2020). Isolation of 2019-nCoV from a Stool Specimen of a Laboratory-Confirmed Case of the Coronavirus Disease 2019 (COVID-19). China CDC Wkly. *2*, 123–124. https://doi.org/10.46234/ccdcw2020.033.

Zhao, X., Li, C., Liu, X., Chiu, M.C., Wang, D., Wei, Y., Chu, H., Cai, J.P., Hau-Yee Chan, I., Kak-Yuen Wong, K., et al. (2021). Human Intestinal Organoids Recapitulate Enteric Infections of Enterovirus and Coronavirus. Stem Cell Rep. *16*, 493–504. https://doi.org/10.1016/J.STEMCR.2021.02.009.

Zhou, J., Li, C., Liu, X., Chiu, M.C., Zhao, X., Wang, D., Wei, Y., Lee, A., Zhang, A.J., Chu, H., et al. (2020). Infection of bat and human intestinal organoids by SARS-CoV-2. Nat. Med. *26*, 1077–1083. https://doi.org/10.1038/s41591-020-0912-6.

Zhu, Z., Lian, X., Su, X., Wu, W., Marraro, G.A., and Zeng, Y. (2020). From SARS and MERS to COVID-19: A brief summary and comparison of severe acute respiratory infections caused by three highly pathogenic human coronaviruses. Respir. Res. *21*, 224. https://doi.org/10.1186/s12931-020-01479-w.

Stem Cell Reports, Volume *19*

Supplemental Information

SARS-CoV-2 tropism to intestinal but not gastric epithelial cells is defined by limited ACE2 expression

Mindaugas Paužuolis, Diana Fatykhova, Boris Zühlke, Torsten Schwecke, Mastura Neyazi, Pilar Samperio-Ventayol, Carmen Aguilar, Nicolas Schlegel, Simon Dökel, Markus Ralser, Andreas Hocke, Christine Krempl, and Sina Bartfeld

Supplementary Material

- **Supplementary Figures**
- **Supplementary Tables and Legends**
- **Supplementary Experimental Procedures**
- **Supplementary References**

Supplementary Figures

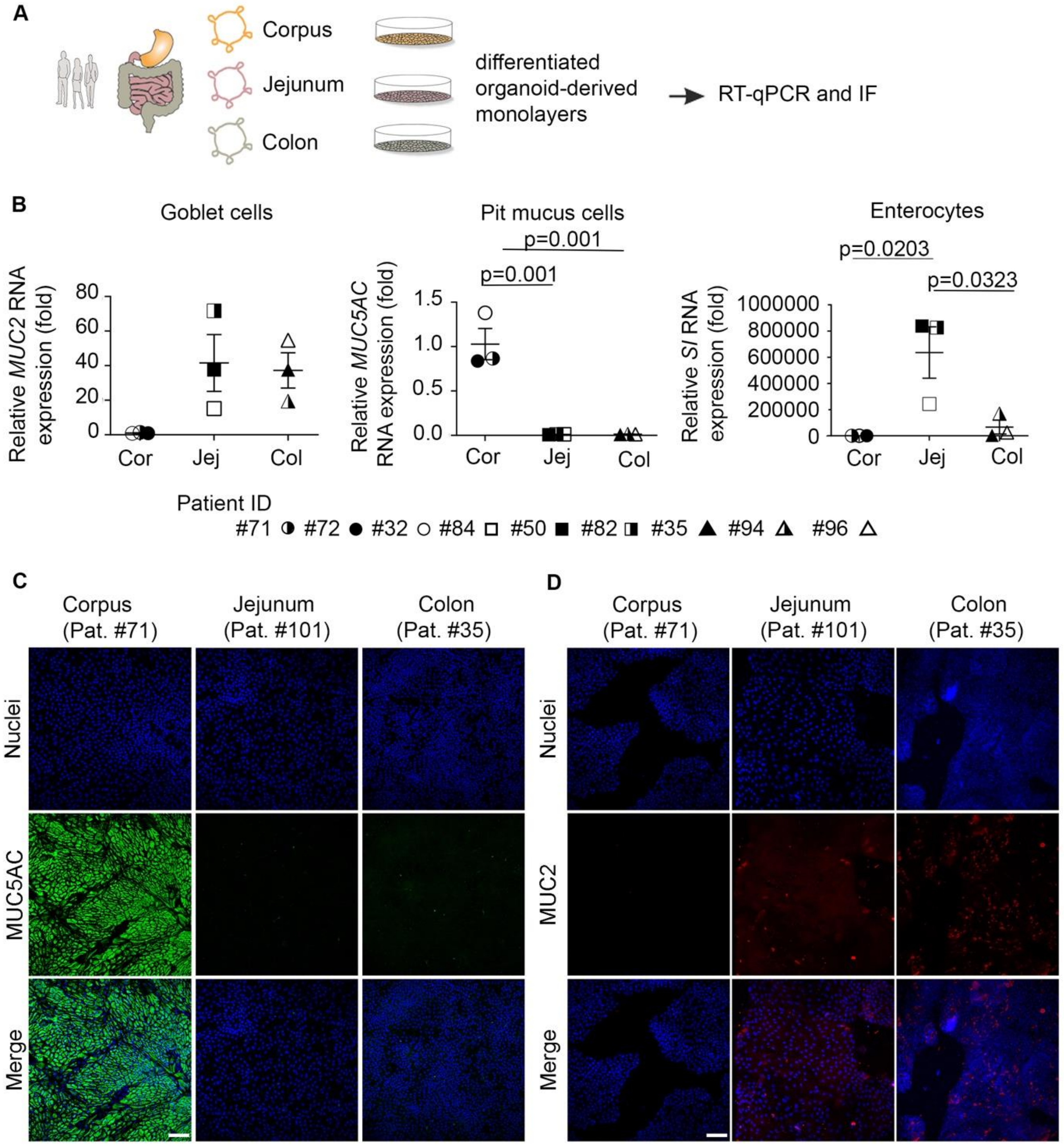

Figure S1: Differentiated GI organoid-derived monolayers express markers of differentiated cells.
A. Schematic setup.
B. Relative mRNA levels of enterocyte (sucrase-isomaltase (*SI*)), goblet cell (*MUC2*), gastric pit cell (*MUC5AC*) markers in RNA isolated from organoid-derived monolayers using RT-qPCR. Data were normalized to *GAPDH* and compared to corpus mRNA levels. Data presented as mean of 3 individual donors ± SEM. Statistical analysis was performed using one-way ANOVA test with Tukey's multiple comparisons test.
C - D. Immunostaining of MUC5AC (green) and MUC2 (red) in differentiated corpus, jejunum and colon organoid-derived monolayers respectively. Nuclei stained with Hoechst 33342. Scale bar is 30 µm.

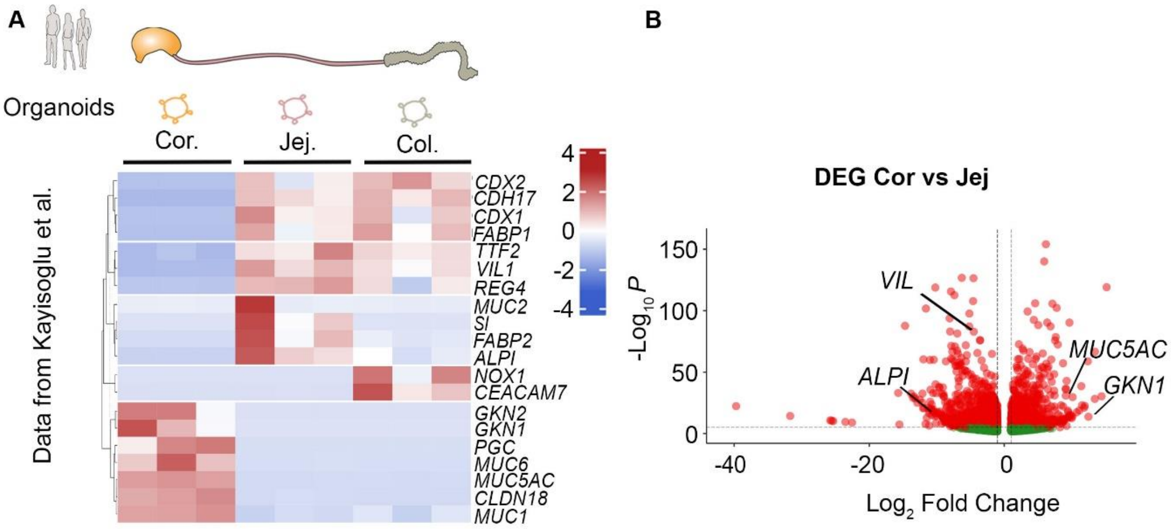

Figure S2: GI organoids show segment-specific expression of GI cell markers.
A. Normalized and scaled expression of selected gastric and intestinal cell markers in corpus, jejunum and colon organoids in GI organoid RNA sequencing data[1].
B. Volcano plot of differentially expressed genes between corpus and jejunum in transcriptomic data[1].

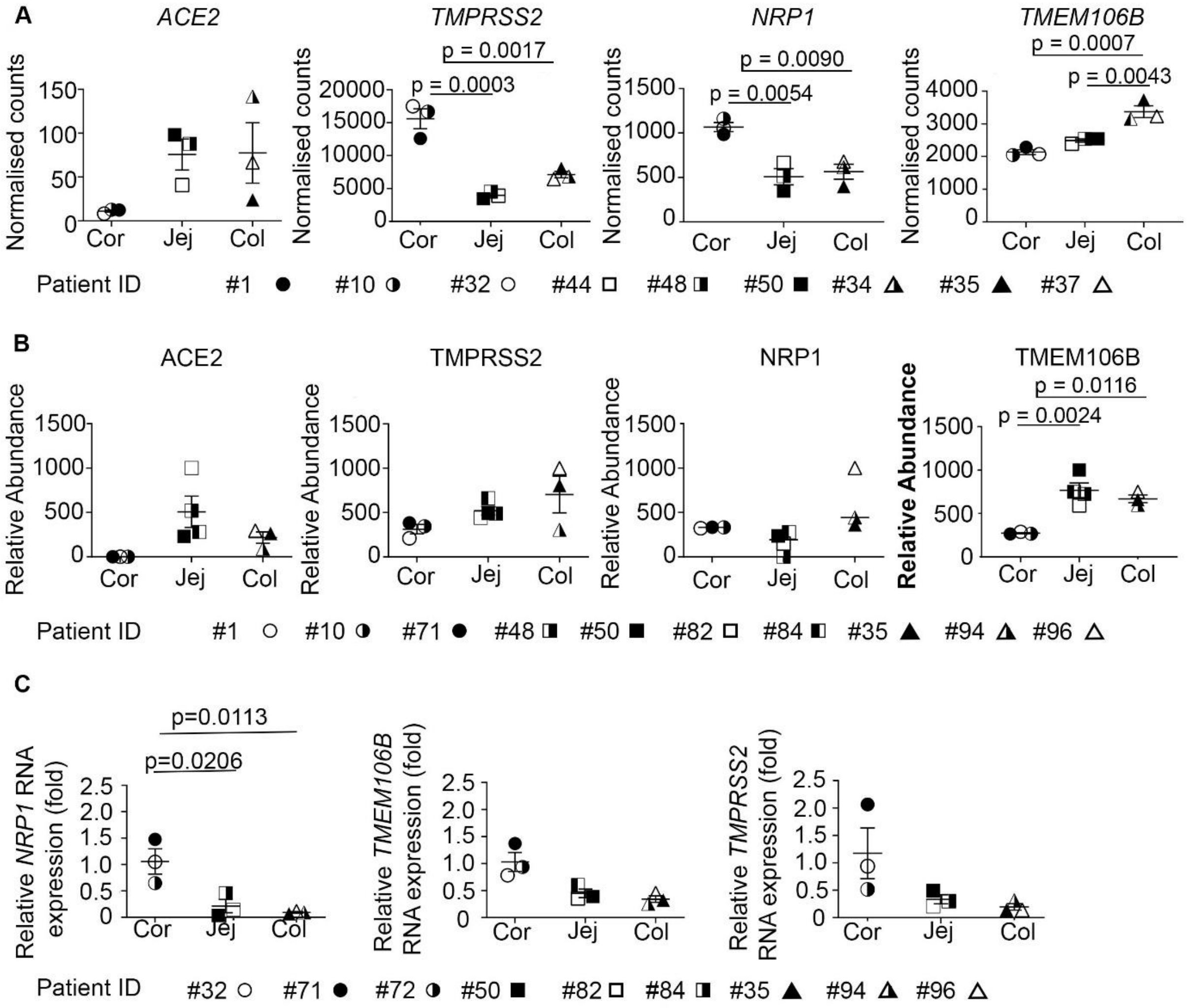

Figure S3: GI expression of SARS-CoV-2 entry factors ACE2, TMPRSS2, NRP1 and TMEM106B in organoids and organoid-derived monolayers.

A. *ACE2, TMPRSS2, NRP1*[2] and *TMEM106B* [3] RNA expression in corpus (Cor), jejunum (Jej), and colon (Col) organoids from published data (Kayisoglu et al. 2021). Data presented as mean ± SEM of 3 individual donors per GI tract segment. Statistical analysis was performed using one-way ANOVA test with Tukey's multiple comparisons test.

B. ACE2, TMPRSS2, NRP1 and TMEM106B protein relative abundance in corpus (Cor), jejunum (Jej) and colon (Col) organoids. Data presented as mean± SEM of 3 individual donors per GI tract segment. Statistical analysis was performed using one-way ANOVA test with Tukey's multiple comparisons test.

C. Relative mRNA levels of *NRP1*, *TMEM106B* and *TMPRSS2* in RNA isolated from differentiated gastrointestinal organoid-derived monolayers quantified using RT-qPCR. Data were normalized to *GAPDH* and compared to average of corpus mRNA levels. Data presented as mean± SEM of 3 individual donors per GI segment. Statistical analysis was performed using one-way ANOVA test with Tukey's multiple comparisons test.

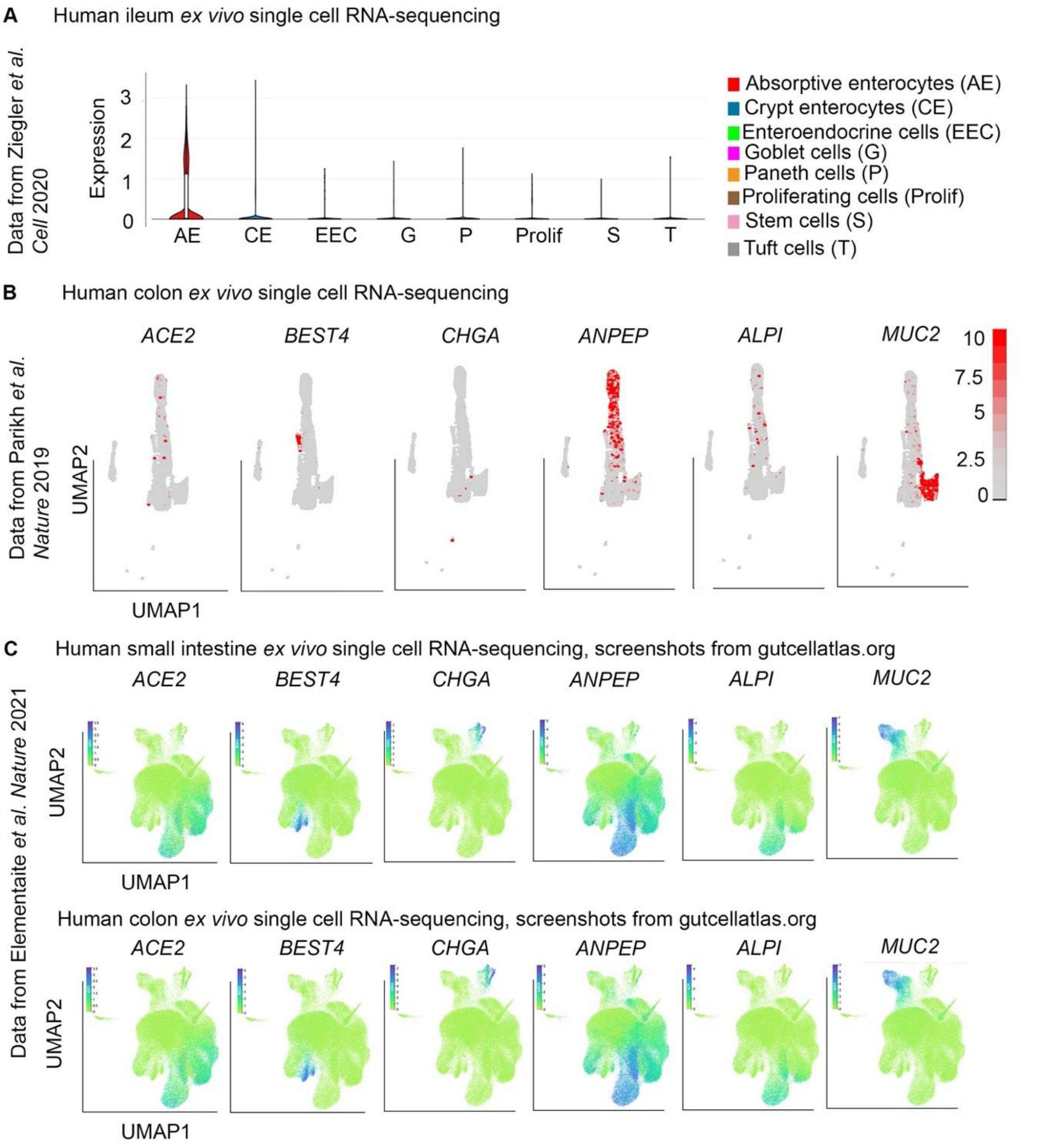

Figure S4: *ACE2* expressing cells overlap with populations expressing markers of enterocytes. A. *ACE2* expression in different cellular populations from human *ex vivo* ileum tissue[4]. Data were visualized using Broad Institute single cell portal (singlecell.broadinstitute.org). The highest expression of *ACE2* is in the subpopulation of absorptive enterocytes, but not enteroendocrine cells. B. Re-analysis of the human *ex vivo* colon tissue data[5] shows expression of *ACE2* in populations that express *ANPEP* and *ALPI*, but not *BEST4*, *CHGA,* or *MUC2*.
C. The expression of *ACE2* co-localized in populations that express *ANPEP* and *ALPI*, but not *BEST4*, *CHGA,* or *MUC2* in human *ex vivo* intestinal and colon tissue[6] . Data were visualized using Gut Cell Atlas (gutcellatlas.org). Specific option selection in gutcellatlas.org: 1) age group: adult. 2) category: Epithelial 3) Region: LargeInt or SmallInt.

Supplementary Tables and Legends

Table S1. Host factors identified by published CRISPR screens. Related to Figure 2B.

See separate spreadsheet.

Table S2. Differentially expressed genes between corpus and jejunum or corpus and colon. Related to Figure 2B.

See separate spreadsheet.

Table S3. Significantly differentially expressed proteins between corpus and jejunum or corpus and colon 3D organoids. Related to Figure 2D.

Protein	Corpus mean	Jejunum mean	Colon mean	p Welch	T-test ind	p Bartlett	pval corrected	Fold change	log2 fold change
DCPS	18,25707	16,63789		8,59E-06	8,59E-06	0,970697	0,004519	3,072006	1,619181
ACE2	0	13,98436		6,27E-05	6,27E-05	0	0,016484	6,17E-05	-13,9844
TMEM106B	14,82227	16,21215		0,000155	0,000155	0,258588	0,027248	0,381598	-1,38987
NELL1	13,34358	0,653031		0,000277	0,000277	0,009806	0,036438	6610,526	12,69055
TCEA3	13,25163	0,78449		0,000351	0,000351	0,060611	0,036921	5662,174	12,46714
NDUFB3	17,79708	18,76953		0,000429	0,000429	0,49187	0,037638	0,509638	-0,97245
NELL1	13,34358		0	1,58E-05	1,58E-05	0	0,008319	10394,84	13,34358
ACE2	0		13,31917	0,000176	0,000176	0	0,046216	9,78E-05	-13,3192

Explanation of column headings:
P Welch: P value of Welch test,
T-test ind: T-test independent sample.
P Bartlett: p value of Bartlett test.
P val corrected: p value corrected using Benjamini-Hochberg test for multiple testing.
Fold change: protein fold change.
log2 fold change: log2 fold protein change.

Table S4. Unique and shared host proteins associated with SARS-CoV-2 infection detected in corpus, jejunum and colon organoid proteome. Related to Figure 2E.

Unique in corpus	Unique in jejunum	Unique in colon	Corpus and colon	Corpus and jejunum	Jejunum and colon
NCAPH2		PRMT7	POLK	NELL1	ACE2
EGR1		HOXB9	TCEA3	PLXNB3	ARPP19
LSM11		CENPW	WEE1	SLC35B4	BRI3
			ZBTB14		CARD10
					SLC25A26

Supplementary Experimental Procedures

List 1: GI tissue donor data.

GI organoid	Patient ID	Age	Gender	Surgical procedure	Carcinoma
Corpus	1	32	F	Sleeve gastrectomy	No
	6	29	F	Sleeve gastrectomy	No
	10	37	F	Sleeve gastrectomy	No
	19	45	M	Sleeve gastrectomy	No
	24	29	F	Sleeve gastrectomy	No
	32	47	F	Sleeve gastrectomy	No
	46	54	F	Gastric carcinoma resection	Yes
	47	75	M	Gastric carcinoma resection	Yes
	58	58	F	Gastric carcinoma resection	Yes
	59	31	M	Sleeve gastrectomy	No
	61	71	F	Gastric carcinoma resection	Yes
	71	82	F	Gastric carcinoma resection	Yes
	72	79	M	Gastric carcinoma resection	Yes
Jejunum	16	-	-	-	-
	20	43	F	Stomach bypass	No
	44	34	F	Stomach bypass	No
	49	64	F	Stomach bypass	No
	50	53	M	Stomach bypass	No
	48	50	M	Stomach bypass	No
	82	41	F	Stomach bypass	No
	84	44	F	Stomach bypass	No
	87	52	F	-	-
	101	32	M	Whipple operation	No
Colon	15	87	M	-	-
	34	52	M	Sigmoid carcinoma resection	Yes
	35	76	F	Hemicolectomy	Yes
	37	74	F	Colon carcinoma resection	Yes
	39	68	F	Coecum carcinoma resection	Yes
	40	66	M	-	-
	94	62	F	Sigmoid resection	No
	96	79	M	Colon carcinoma resection	Yes

SARS-CoV-2 infection

Gastrointestinal monolayers were inoculated with 50 µl of SARS-CoV-2[7], containing $1x10^6$ plaque-forming units per well, and incubated for 1h (37°C, 5% CO2) with periodical shaking. After incubation, the inoculum was removed, and monolayers were washed 3 times with PBS. Monolayers were incubated in differentiation media for 24 h before collection.

SARS-CoV-2 N protein IF staining in gastrointestinal monolayers

SARS-CoV-2 protein expression was evaluated with SARS N protein staining infected monolayers. After infection, cells were fixed with 4 % paraformaldehyde for 30 min at RT, washed with PBS two times, and permeabilized with 0.5 % Triton X-100 in PBS for 15 min. Mouse anti-SARS-CoV N (1:1000, MM05, Sino Biologicals) was diluted in PBS and incubated at RT for 1h. The secondary antibody (1:500 goat anti-mouse Alexa Fluor-488, A-11001, Thermo Fisher Scientific) was diluted in PBS and incubated at RT for 1h. Nuclei were stained with Hoechst 33342 (1:5,000; H3570, Life Technologies). Images of infected cells were acquired with EVOS™ FL Digital Inverted Fluorescence Microscope (Invitrogen).

SARS-CoV-2 RNA isolation and viral titer quantification using RT-qPCR

Gastrointestinal monolayers were lysed using TRI Reagent and RNA was isolated following the protocol of Direct-zol RNA MiniPrep (R2052, Zymo Research). cDNA was generated using random

primers (48190011, Life Technologies) and M-MuLV reverse transcriptase (M0253, New England Biolabs) according to the manufacturer's protocol. Quantitative reverse transcription PCR was carried out using SYBR Green Supermix (172-5270, BioRad) in a CFX96 Touch Real-Time PCR detection system using Bio-Rad CFX Manager software (BioRad). Viral titers were normalized to non-infected control using the ΔΔCt values method and normalized to the *18S* gene. List of all primers used in the study are listed below in the section RT-qPCR.

Plaque assay

Plaque assay was carried out using Vero TMPRSS2 cells. Cells were cultured in DMEM w/Glutamax (12634028, Thermo Fisher) supplemented with 10% FCS (S0615, Biochrom) and 1% P/S (15140122, Thermo Fisher). The supernatant of infected cells was serially diluted before the inoculation of Vero TMPRSS2 cells. Inoculated cells were incubated at 37°C for 1 hour with periodical shaking. The cells were overlayed with 1ml of 0.6 % methylcellulose overlay and incubated for 4 days at 37°C and 5% CO_2. Plaques were fixed with 20% Methanol solution supplemented with 0.2 % Crystal violet and incubated overnight. The total number of plaques per well was counted and used to calculate PFU/ml.

GI organoid culture

Organoids were cultured in Advanced DMEM/F12 supplemented with GlutaMAX, 10 mM HEPES (AD++), B27 (all Thermo Fisher), 50% Wnt, 10% R-spondin, 10% noggin conditioned media, 1.25 mM N-acetyl-cysteine (Sigma-Aldrich), 100 ng/mL Primocin (Invivogen), 1 nM (gastric) or 10 nM (intestinal) Gastrin I (Tocris), 50 ng/mL EGF (Peprotech), 2 µM (gastric) or 0.5 µM TGF-beta inhibitor (Tocris), 100 ng/mL FGF-10 (only gastric, Peprotech), 50 ng/mL IGF-1 (only intestinal, Biolegend), 50 ng/mL FGF-basic (only intestinal, Biolegend).

List of 3D organoid expansion and 2D organoid-derived monolayer differentiation medium composition.

**3D organoid expansion media composition**		
Medium components	Gastric media	Colon/Jejunum media
AD DMEM /F12 (12634028, Thermo Fisher Scientific)	30% of total volume	30% of total volume
1X GlutaMAX-I (35050-038, Thermo Fisher Scientific)		
10mmol/L HEPES (15630056, Thermo Fisher Scientific)		
WNT conditioned media	50% of total volume	50% of total volume
R-spondin conditioned media	10% of total volume	10% of total volume
Noggin conditioned media	10% of total volume	10% of total volume
B27 (12587010, Thermo Fischer Scientific)	1X	1X
N-acetyl-cysteine (A9165-25G, Sigma-Aldrich)	1.25mM	1.25mM
Primocin (Ant-pm-1, Invivogen)	100 ng/mL	100 ng/mL
Gastrin-I (3006, Tocris)	1 nM	10 nM
EGF (AF-100-15, Peprotech)	50 ng/mL	50 ng/mL
FGF-10 (100-26, Peprotech)	100 ng/mL	-
A83-01 (A-83-01, Tocris)	2 µM	0.5 µm
IGF-1 (590904, Biolegend)	-	100 ng/mL
FGF-basic (C100-18B, Peprotech)	-	50 ng/mL
**2D organoid-derived monolayer differentiation media composition**		
Medium components	Gastric DIF media	Colon/ Jejunum DIF media
AD DMEM /F12 (12634028, Thermo Fisher Scientific)	80% of total volume	90% of total volume
1X GlutaMAX-I (35050-038, Thermo Fisher Scientific)		
10mmol/L HEPES (15630056, Thermo Fisher Scientific)		

WNT conditioned media	-	-
R-spondin conditioned media	10% of total volume	5% of total volume
Noggin conditioned media	10% of total volume	5% of total volume
B27 (12587010, Thermo Fischer Scientific)	1X	1X
N-acetyl-cysteine (A9165-25G, Sigma-Aldrich)	1.25mM	1.25mM
Primocin (Ant-pm-1, Invivogen)	100 ng/mL	100 ng/mL
Gastrin-I (3006, Tocris)	1 nM	10 nM
EGF (AF-100-15, Peprotech)	50 ng/mL	50 ng/mL
FGF-10 (100-26, Peprotech)	100 ng/mL	-
A83-01 (A-83-01, Tocris)	2 µM	0.5 µm

TCID50 assay

TCID50 assay of supernatant of infected gastrointestinal monolayers was carried out using Vero TMPRSS2 cells. Cells were cultured as described above. The supernatant of infected cells was serially diluted before the inoculation of Vero TMPRSS2 cells. Cells were inoculated with 80 µl of diluted inoculum and incubated for 24h at 37°C and 5% CO2. Cells were fixed with 4% PFA and proceeded to SARS-CoV-2 N protein staining. SARS-CoV-2 positive cells were images and used to determine TCID50.

RT-qPCR for gastrointestinal tissue and organoid-derived monolayers

Total RNA was isolated from gastrointestinal tissue and monolayers using RNeasy Mini Kit (74106, Qiagen) according to the manufacturer's instructions. cDNA was generated using random primers (48190011, Life Technologies) and M-MuLV reverse transcriptase (M0253, New England Biolabs) according to the manufacturer's instructions for monolayers. cDNA for tissue isolated RNA was generated using TaqMan™ Reverse Transcription kit (N8080234, Applied Biosystems). The quantification of RNA levels for tissue origin cDNA was done using SensiFAST™ SYBR® No-ROX Kit (98020, Bioline). Quantitative reverse transcription PCR analysis for monolayer samples was performed using SYBR Green Supermix (172-5270, BioRad) following the manufacturer's instructions, in a CFX96 Touch Real-Time PCR detection system using Bio-Rad CFX Manager software (BioRad) Primers used in this study are listed below. The expression of SARS-CoV-2 entry factors and gastrointestinal cell markers was analyzed using the ΔΔCt method.

List of RT-qPCR assay primers.

Gene	Sequence
GAPDH	(F) 5-CTCTCTGCTCCTCCTGTTCGAC-3
	(R) 5-TGAGCGATGTGGCTCGGCT-3
ACE2	(F) 5-TCAGAACCCTGGACCCTAGC-3
	(R) 5- GTTCTGGTCTTTCAGCCAGGT-3
IFNL2/3	(F) 5-GCCACATAGCCCAGTTCAAG-3
	(R) 5-TGGGAGAGGATATGGTGCAG-3
SARS-CoV-2 N	(F) 5- GCCTCTTCTGTTCCTCATCAC-3
	(R) 5- AGACAGCATCACCGCCATTG-3
18S	(F) 5-TGTGCCGCTAGAGGTGAAATT-3
	(R) 5-TGGCAAATGCTTTCGCTTT-3
SARS-CoV-2 RdRp	(F) 5- GTGARATGGTCATGTGTGGCGG-3
	(R) 5-CARATGTTAAASACACTATTAGCATA-3
IFNL1	(F) 5- GCAGGTTCAAATCTCTGTCAC -3
	(R) 5- AAGACAGGAGAGCTGCAACTC -3
TMPRSS2	(F) 5- CTTTGAACTCAGGGTCACCA -3
	(R) 5- TAGTACTGAGCCGGATGCAC -3
NRP1	(F) 5-GCCACAGTGGAACAGGTGAT -3
	(R) 5-ATGACCGTGGGCTTTTCTGT-3
TMEM106B	(F) 5- TCCACGACCCTGTCCTCG -3

	(R) 5- AGACTTTCCCATGTCGGCAC -3
MUC5AC	(F) 5- CTTCTCAACGTTTGACGGGAAGC-3
	(R) 5- CTTGATCACCACCACCGTCTG -3
MUC2	(F) 5- CGTCCGTCTCCAACATCACC -3
	(R) 5- CACCCTGGTCTCATTGCGAG -3
SI	(F) 5- AATCCTTTTGGCATCCAGAT -3
	(R) 5- GCAGCCAAGAATCCCAAAT -3

Immunofluorescence staining

Gastrointestinal monolayers were seeded on µ-Slide 8 Wells (80826, Ibidi) and cultured under differentiation conditions for 4 days. After differentiation, cells were fixed with 4 % paraformaldehyde for 20 min at RT, permeabilized with 0.5 % Triton X-100 in PBS for 15 min, and then blocked with 1% BSA for 1 h. Cells were stained with ACE2 (1:100, AF933, R&D Systems) MUC5AC (1:100, MA5-12178, Thermo Fischer Scientific), and MUC2 (1:400, sc-15334, Santa Cruz Biotechnology) diluted in blocking buffer overnight at 4°C. Gastrointestinal monolayers were incubated with corresponding secondary antibodies donkey anti-goat Alexa Fluor-488 (1:500, A-11055, Thermo Fisher Scientific), donkey anti-rabbit Alexa Fluor-594(1:500, A-21207, Invitrogen), goat anti-mouse Alexa Fluor-488 (1:500, A-11001, Thermo Fisher Scientific) in blocking solution and incubated at RT for 2 h. Nuclear DNA was stained with Hoechst 33342 (1:5,000; H3570, Life Technologies). Gastrointestinal monolayers were imaged using a Leica SP5 laser scanning confocal microscope (Leica Microsystems) and images were analyzed with Fiji open-source software.

Immunohistochemistry and *In situ* hybridization

Gastrointestinal tissue biopsy samples were fixed using formalin 10% (09122, Neogen) and embedded in paraffin blocks. Embedded tissue sections (5 µm) were routinely processed for histology and immunofluorescence staining as described before[8, 9,10]. Tris-EDTA (10 mM Tris base, 1 mM EDTA solution, pH 9.0) buffer was used for antigen retrieval. Gastrointestinal tissue biopsy samples were probed with MUC2 (1:400, sc-15334, Santa Cruz Biotechnology) and ACE2 (1:100, AF933, R&D Systems) at 4°C overnight, followed by incubation with corresponding secondary antibodies (1:2000, Alexa Fluor488 and Alexa Fluor647, Invitrogen). Nuclear DNA was counter-stained using DAPI (Sigma Aldrich). To examine ACE2 RNA expression in gastrointestinal tissue, fluorescence RNA ISH was carried out using RNAscope Multiplex Fluorescent Reagent Kit v2 (Advanced Cell Diagnostics, Bio-Techne) using available ACE2 probe (probe Hs-ACE2-C2; Advanced Cell Diagnostics), according to the manufacturer's protocol. Immunofluorescence images of stained gastrointestinal tissue were acquired with LSM 780 confocal microscope [(objectives: Plan Apochromat 40x/1.40 oil DIC M27 and Plan Apochromat 10x/0.3 DIC M27), Carl-Zeiss, Jena, Germany]. To reveal tissue and cell morphology, images were combined with Differential Interference Contrast (DIC). All image sets were acquired using equal configurations. Images were processed using ZEN 2012.

Western blot

Organoids were lysed organoids were lysed in RIPA buffer (89900, Thermo Scientific) with protease inhibitor cocktail (11697498001, Roche) (1:50) on ice, followed by sonication. Proteins were separated on SDS–PAGE gels and transferred to the nitrocellulose membrane. Membrane were probed with following antibodies: anti-ACE2 (1:100, AF933, R&D Systems), anti-Tubulin (1:3,000, A2228, Sigma-Aldrich), anti-mouse HRP (1:5000, ab97040, Abcam), anti-goat HRP (1: 5000, sc-2354, Santa Cruz Biotechnology). Protein bands were visualized using luminol (0,25mg/mL, A8511-5G, Sigma-Aldrich) and p-coumaric acid (1.1 mg/mL, sc-215648A, Santa Cruz Biotechnologies) solution. The images were taken using an ImageQuant LAS 4000 CCD camera (GE Healthcare).

RNA-seq analysis

The previously published 3D organoids RNA sequencing data (GEO access number GSE127938) was used to examine the expression of known host factors for SARS-CoV-2 infection using R (4.1.2) and Bioconductor packages[1]. The list of known SARS-CoV-2 infection host factors was compiled using top hits from previously published studies and after filtration 749 genes were used for further

analysis[11-15] (see Table S1). The raw counts of organoid data were normalized using standard DESeq2 analysis and visualized as previously described[1]. The differential expression analysis between corpus, jejunum, and colon organoids was carried out using Benjamini-Hochberg adjusted p-value below 0.05 as the cut-off value. The differential expression data was visualized using the EnhancedVolcano package.

Single cell-RNA sequencing (sc-RNA-seq) data analysis

Publicly available sc-RNA sequencing data were analyzed for the expression of *ACE2, BEST4, CHGA, MUC2, ALPI,* and *ANEP*. Human *ex vivo* small intestine and colon tissue data was analyzed using a gut cell atlas (gutcellatlas.org) containing sc-RNA-seq data of adult intestinal epithelium[6]. Data was visualized using the following settings: age group: adult, category: epithelial, region: largeInt or smallInt. Also, human *ex vivo* ileum sc-RNA-seq data[4] were examined for cell type-specific expression of ACE2 using the Broad Institute single-cell portal (Singlecell. broadinstitute.org). Furthermore, available human *ex vivo* colonic epithelium sc-RNA-seq data[5] (GEO access number GSE116222). was used to visualize the expression of *ACE2, BEST4, CHGA, MUC2,* and *ALPI* as well as *ANEP* using Seurat R package V4[16].

ACE2 overexpression in corpus organoids

ACE2 overexpression in corpus organoids was induced by using a commercially available lentivirus construct of ACE2 (79944, BPS Bioscience). Organoids (patient ID #71) were collected and washed with AD++, centrifugated, and resuspended in TrypLE Express (Gibco). Organoids were incubated for 10 min at 37°C with occasional resuspension with P1000 pipette. Cells were washed with AD++ and pelleted. Cells were resuspended in AD++ supplemented with polybrene (8µg/mL, TR1003G, Thermo Fisher Scientific), RHOKi (10 µM, Y-27632, Sigma-Aldrich) and nicotinamide (10 mM, N0636, Sigma-Aldrich) and seeded into 48-well plate coated with 80µL mix of Matrigel and AD++ (1:2). Cells were transduced with MOI2 of lentivirus and incubated for 16h at 37°C. Following the incubation wells were washed with AD++ and TrypLE Express to collect most of the cells. The collected cells were washed with AD++ and seeded into 3 wells in Matrigel domes and overlayed with complete gastric medium supplemented with Wnt Surrogate-Fc Fusion Recombinant Protein (PHG0403, Gibco) and RHOKi. Organoids were selected with puromycin (1 µg/mL) 48h post-seeding into Matrigel. The puromycin selection was continued for 7 days with full gastric medium supplemented with a Wnt surrogate. Surviving organoids were expanded and characterized for ACE2 expression in differentiated 2D monolayers using immunofluorescent staining.

Mass spectrometry sample preparation

Gastrointestinal organoids were cultured for 8 days after splitting in gastric and EIF expansion medium before collection for protein extraction. GI organoids were lysed in RIPA buffer (Thermo Scientific, 89900) with protease inhibitor cocktail (11697498001, Roche) on ice. Cell debris was removed (2500 rpm for 5 min) and protein concentration was determined (Pierce, 23225). A volume corresponding to 50 µg protein was transferred and topped up to 60 µl with RIPA buffer. Lysates were processed manually using the SP3 protocol as previously described[17] with one-step reduction and alkylation. Briefly, 10 µl of reduction and alkylation buffer (40 mM TCEP, 160 mM CAA, 200 mM ABC, 4% SDS) were added, and samples were incubated at 56 °C for 30 min and cooled to RT. To bind the proteins, 500 µg of paramagnetic beads (1:1 ratio of hydrophilic/hydrophobic beads (GE Healthcare, PN 45152105050250, 65152105050250) were added and proteins were precipitated by adding ACN to a final concentration of 70%. Samples were washed twice with 80% EtOH and once with 100% ACN before reconstitution in 35 µl 100 mM ABC. Samples were digested overnight at 37 °C with Trypsin/Lys-C mix (Promega, V5072) at a protein: enzyme ratio of 50:1 (w/w). The reaction was stopped by adding formic acid to a final concentration of 0.1%. The resulting tryptic peptides were purified using C18-based (Affinisep, AttractSPE) stage tips as described preveliously[18] and dried before resuspension in 0.1% formic acid. The peptide concentration was determined (Pierce, 23290), insoluble particles were removed by centrifugation, samples were transferred to a new plate, and frozen at -80 °C until analysis by LC-MS.

Liquid chromatography-mass spectrometry

LC–MS analysis was conducted on an EVOSEP One system coupled to a Bruker TimsTOF PRO2 mass spectrometer. Five hundred nanograms of sample material were loaded onto the Evotip according to the manufacturer's protocol. Liquid chromatography was performed using the EVOSEP 15 SPD LC method (88 min gradient) with an EV1137 performance column (15 cm × 150 µm, 1.5 µm) at 40 °C, coupled to a 10 µm Zero Dead Volume Captive Spray Emitter (Bruker #1865691). For acquisition in dia-PASEF mode, the acquisition scheme covered the mass range m/z 400–1,201 and ion mobility range 1/K0 0.6–1.6, using 16 frames, with two precursor isolation windows per frame (m/z 26 window width, m/z 1.0 overlap between adjacent windows). Accumulation and ramp times were set to 100 ms. The mass spectrometry proteomics data have been deposited to the ProteomeXchange Consortium via the PRIDE[19] partner repository with the dataset identifier PXD044789.

Proteomics mass spectrometry data analysis

The raw proteomic mass spectrometry data was processed with *DIA-NN* version *1.8* [20] using default settings. MS1 and MS2 mass accuracies were fixed to 20 ppm and a spectral library was generated from the *H. sapiens* Proteome UP000005640_9606 downloaded from UniProt[21]. All following proteomics analysis steps were performed using Python 3.9.7. Precursors were filtered for q-values < 0.01 (on sample, global, and library levels), and only quantified proteotypic precursors were considered for analysis. Afterward, precursors were present in less than 2/3 of the samples, and proteins quantified by less than 2 precursors were excluded. Following this, precursor intensity distributions were normalized using median polish normalization on precursors present in 90% of all samples, where every precursor was multiplied by a sample-specific factor given by the ratio of the median of the sample medians of precursor intensities and the median of precursor intensities in a given sample. For imputing missing data, we first distinguished missingness not at random from missingness at random by counting the number of precursors originating from every protein. If no precursor for a given protein was identified, the precursor value was set to zero, otherwise, the *k-nearest-neighbors* algorithm from the *scikit-learn* package *version 1.2.2* [22] was applied to log2-scaled precursors using *k=2*. Last, precursors were summarized to proteins using the MaxLFQ algorithm as implemented in QuantUMS[23].
Sample quality was assessed during all data processing steps by comparing the numbers of identified precursors, precursor intensity distributions, normalization factors obtained from median polish normalization, and statistical parameters from the DIA-NN report such as signal and median mass accuracies on MS1 and MS2 as well as the average number of missed tryptic cleavages. Additionally, technical quality was assessed by comparing samples to three sample pools containing aliquots of all biological samples. One sample (Corpus #72) did not meet the quality of the other samples and was therefore excluded. Finally, the data processing pipeline was then executed again only on samples satisfying the quality standards excluding the quality control samples.
Clustermaps with proteomic profile correlations and hierarchical clustering of samples were created with the clustermap function from the Python *seaborn* package version *0.11.2* [24]. For differential analysis between corpus, colon, and jejunum organoids Welch tests from the S*ciPy* package version *1.10.1* on log2 protein MaxLFQ abundances with Benjamini-Hochberg multiple testing correction from the *statsmodels* package version *0.13.5* [25] were applied, and plotted against protein mean fold changes. Differentially expressed genes were identified as proteins with absolute mean log2 fold changes between two organoid groups larger than 1 as well as an adjusted p-value below 0.05.

Differential detection of proteins (Figure 2 G) was performed on data before the exclusion of precursors with excessive missing values and before the exclusion of proteins identified by less than two precursors. A protein was considered detected as long as it was present in at least one organoid sample per group (corpus, colon, or jejunum). The relative abundance of selected proteins (Figure S3B) was calculated as follows – Relative abundance = (MaxLFQ (protein of interest) / maximal detected value protein of interest (MaxLFQ)).

Statistics

Experimental results are presented as mean ± standard error of the mean (SEM) of individual patients. There available organoid lines are listed in figure legends as well as statistical tests used for the analysis. Prism Software (v8, GraphPad) was used to carry out statistical analysis using the threshold of significance at $p < 0.05$.

Supplementary References:

1. Kayisoglu, O. et al. (2021). Location-specific cell identity rather than exposure to GI microbiota defines many innate immune signalling cascades in the gut epithelium. Gut. 70(4):687-697. doi: 10.1136/gutjnl-2019-319919.

2.Cantuti-Castelvetri, L. et al. (2020). Neuropilin-1 facilitates SARS-CoV-2 cell entry and infectivity. Science, 370(6518), pp. 856–860. doi: 10.1126/science.abd2985.

3. Baggen, J. et al. (2023). TMEM106B is a receptor mediating ACE2-independent SARS-CoV-2 cell entry. Cell. Aug 3;186(16):3427-3442.e22. doi: 10.1016/j.cell.2023.06.005.

4. Ziegler, C. G. K. et al. (2020).SARS-CoV-2 Receptor ACE2 Is an Interferon-Stimulated Gene in Human Airway Epithelial Cells and Is Detected in Specific Cell Subsets across Tissues. Cell. 181(5):1016-1035.e19. doi: 10.1016/j.cell.2020.04.035.

5. Parikh, K. et al. (2019). Colonic epithelial cell diversity in health and inflammatory bowel disease. Nature, 567(7746):49-55.doi: 10.1038/s41586-019-0992-y.

6. Elmentaite, R. et al. (2021). Cells of the human intestinal tract mapped across space and time. Nature, 597(7875):250-255. doi: 10.1038/s41586-021-03852-1.

7. Peterhoff et al. (2021). A highly specific and sensitive serological assay detects SARS-CoV-2 antibody levels in COVID-19 patients that correlate with neutralization. Infection. 49(1):75-82. doi: 10.1007/s15010-020-01503-7.

8. Berg, J. et al,. (2017). Tyk2 as a target for immune regulation in human viral/bacterial pneumonia. Eur Respir J 50(1):1601953. doi: 10.1183/13993003.01953-2016.

9. Hocke A.C. et al (2013). Emerging human Middle East respiratory syndrome coronavirus causes widespread infection and alveolar damage in human lungs. Am J Respir Crit Care Med. 188(7):882-6. doi: 10.1164/rccm.201305-0954LE.

10. Hönzke, K. et al. (2022). Human lungs show limited permissiveness for SARS-CoV-2 due to scarce ACE2 levels but virus-induced expansion of inflammatory macrophages. Eur Respir J 60(6):2102725. doi: 10.1183/13993003.02725-2021

11.Wang, R. et al. (2021). Genetic Screens Identify Host Factors for SARS-CoV-2 and Common Cold Coronaviruses. Cell. 184(1):106-119.e14. doi: 10.1016/j.cell.2020.12.004.

12. Schneider, W. M. et al. (2021). Genome-Scale Identification of SARS-CoV-2 and Pan-coronavirus Host Factor Networks. Cell. 184(1):120-132.e14. doi: 10.1016/j.cell.2020.12.006.

13. Daniloski, Z. et al. (2020).Identification of required host factors for SARS-CoV-2 infection in human cells.Cell. 184(1):92-105.e16. doi: 10.1016/j.cell.2020.10.030.

14. Schmidt, N. et al. (2020). The SARS-CoV-2 RNA–protein interactome in infected human cells. Nat Microbiol. 6(3):339-353. doi: 10.1038/s41564-020-00846-z.

15. Wei, J. et al. (2020). Genome-wide CRISPR Screens Reveal Host Factors Critical for SARS-CoV-2 Infection. Cell. 184(1):76-91.e13. doi: 10.1016/j.cell.2020.10.028.

16. Hao, Y. et al. (2021). Integrated analysis of multimodal single-cell data. Cell. 184(13):3573-3587.e29. doi: 10.1016/j.cell.2021.04.048.

17. Müller T, et al., (2020). Automated sample preparation with SP3 for low-input clinical proteomics. J. Mol Syst Biol. 16(1):e9111.doi: 10.15252/msb.20199111.

18. Rappsilber J, Mann M, Ishihama Y. (2017). Protocol for micro-purification, enrichment, pre-fractionation and storage of peptides for proteomics using stagetips. Nature Protocols. 2(8):1896-906. doi: 10.1038/nprot.2007.261.

19. Perez-Riverol Y. et al., (2022). The PRIDE database resources in 2022: A Hub for mass spectrometry-based proteomics evidences. Nucleic Acids Res 50 (D1):D543-D552. doi: 10.1093/nar/gkab1038.

20. Demichev, V. et al., (2020). DIA-NN: neural networks and interference correction enable deep proteome coverage in high throughput. Nature methods, 17(1):41-44. doi: 10.1038/s41592-019-0638-x

21. https://www.uniprot.org/proteomes/UP000005640, Access date: 31.01.2022, 12:00 CET.

22. Pedregosa et al., (2011). Scikit-learn: Machine Learning in Python. JMLR 12, pp. 2825-2830.

23. Kistner, F. et al. (2023). QuantUMS: uncertainty minimisation enables confident quantification in proteomics. bioRxiv, 2023-06.

24. Waskom, M. L. (2021). Seaborn: statistical data visualization. Journal of Open Source Software, 6(60), 3021.

25. Seabold, Skipper, and Josef Perktold. (2010). statsmodels: Econometric and statistical modeling with python. Proceedings of the 9th Python in Science Conference.
